# Long Non-coding RNA ENST00000453774.1 Confers an Inhibitory Effect on Renal Fibrosis by Inhibiting miR-324-3p to Promote NRG1 Expression

**DOI:** 10.3389/fcell.2021.580754

**Published:** 2021-11-19

**Authors:** Shumei Tang, Gong Xiao, Qiongjing Yuan, Wei Lin, Xiangning Yuan, Xi Fang, Tianci Deng, Xiangcheng Xiao

**Affiliations:** ^1^Department of Nephrology, Xiangya Hospital, Central South University, Changsha, China; ^2^Department of Pathology, Xiangya Hospital, Central South University, Changsha, China

**Keywords:** long non-coding RNA ENST00000453774.1, autophagy, renal fibrosis, microRNA-324-3p, NRG1, PI3K/AKT signaling pathway

## Abstract

Progressive or chronic renal diseases arise from a process of destructive renal fibrosis. Therefore, the molecular basis of renal fibrosis has attracted increasing attention. In this investigation, we set out to elucidate the potential interaction among long non-coding RNA ENST00000453774.1 (lncRNA 74.1), microRNA-324-3p (miR-324-3p), and NRG1, and to investigate their roles in the context of cellular autophagy and renal fibrosis. We collected 30 renal fibrosis tissue samples for analysis. In other studies, HK-2 cells were stimulated with TGF-β1 to induce a cell model of renal fibrosis, followed by alteration on the expression of lncRNA 74.1, miR-324-3p, or NRG1, or by the addition of AKT activator SC79 in the HK-2 cells. The expression levels of lncRNA 74.1, miR-324-3p, NRG1, autophagy-related proteins (ATG5, ATG7, LC3II/I, and P62), and the corresponding fibrosis markers (Collagen I, Fibronectin, and α-SMA) were subsequently determined using various assay methods. In addition, the proportion of LC3 positive cells and number of autophagosomes were recorded. Results revealed that lncRNA 74.1 and NRG1 were poorly expressed and miR-324-3p was highly expressed in renal fibrosis tissues and modeled cells. LncRNA 74.1 could bind to miR-324-3p, which led to upregulated NRG1 expression and inhibition of the PI3K/AKT signaling pathway. Meanwhile, overexpression of lncRNA 74.1 or down-regulation of miR-324-3p increased the levels of ATG5, ATG7, LC3II, and LC3I, and decreased levels of P62, Collagen I, Fibronectin, and α-SMA, accompanied by elevated proportions of LC3 positive cells and autophagosomes. Findings concur in showing that lncRNA 74.1 could induce cellular autophagy and alleviate renal fibrosis by regulating the miR-324-3p-mediated NRG1/PI3K/AKT axis. This axis may thus present a potential molecular target in renal fibrosis treatment.

## Introduction

Renal fibrosis is a fibrogenic process, which is a common pathological finding in almost all kinds of chronic and progressive kidney diseases. Typically, the deposition of fibrotic matrix after a renal injury can prove beneficial during the early stages of tissue repair, followed by gradual resorption as the injury is resolved (Humphreys, 2018). Pathologically, renal fibrosis manifests upon chronic and continuous fibrotic matrix deposition, which interferes with tissue architecture and finally compromises the physiological renal function. Recent statistical reports indicate that chronic kidney disease (CKD) affects over 20 million adults in America. Further adding to the burden of disease, the increasing incidence of CKD in younger populations is a cause for concern (Coresh et al., 2007; Murphy et al., 2016). Clinically, several pathological factors are analyzed to assess the degree of renal fibrosis, such as graft quality, inflammation, and genetic factors (Vanhove et al., 2017). Meanwhile, the currently used treatment protocols for renal fibrosis include the induction of endogenous antifibrotic factor expression or administration of anti-inflammatory medications (Liu, 2006; Meng et al., 2016). On a cellular scale, accumulation of excessive connective matrix proteins is regarded to be central in the development of renal fibrosis. Moreover, collagen 1, which is primarily secreted by myofibroblast cells, is known to be abundantly present in the matrix in renal fibrosis (Kramann et al., 2013). Therefore, we can speculate that a treatment interfering in the differentiation of myofibroblasts could serve as an alternative treatment for renal fibrosis.

Research conducted around the world has highlighted transforming growth factor-β (TGF-β) as the chief regulator of renal fibrosis. Among the several TGF-β isoforms, TGF-β1 notably possesses the ability to initiate renal fibrosis either by canonical (SMAD-based) or non-canonical (non-SMAD-based) signaling pathways, by activating the myofibroblasts to produce excessive extracellular matrix (ECM) production and/or inhibiting ECM degradation (Lan, 2011). On the other hand, microRNAs (miRNAs) are short single-stranded RNA, not exceeding 22 nucleotides and extensively-participate in cellular regulation in a wide range of eukaryotes (Leung, 2015). A comprehensive interaction between TGF-β1 and miRNAs has been previously demonstrated to regulate the progression of renal fibrosis (Meng et al., 2016). For instance, there was significant upregulation of miR-21 in CKD, where its expression was positively correlated with the stage of renal fibrosis and the prognosis of patients’ treatment (McClelland et al., 2015). Meanwhile, long non-coding RNAs (lncRNAs) are another group of regulating RNAs, which exhibit vital actions in a wide range of cellular processes. Furthermore, findings obtained in our previous study suggested that lncRNA 74.1 down-regulation can contribute to renal fibrosis, while its overexpression might represent a novel anti-fibrotic treatment in renal diseases (Xiao et al., 2019). Next-generation sequencing in a previous study further documented that some lncRNAs could be regulated by TGF-β/Smad3 signaling in the kidneys (Zhou et al., 2014). PI3K/AKT signaling pathway is the upstream regulator of the mTOR-signaling pathway, a critical process in cellular metabolism; an existing study elucidated the involvement of the PI3K/AKT/mTOR axis with renal fibrosis (Dou et al., 2019). However, it remains to be established whether other regulators may co-ordinate with the PI3K/AKT signaling pathway to affect renal fibrosis.

In the present study, we elucidated a new regulatory mechanism combining lncRNA and miRNA to ameliorate renal fibrosis. Our findings revealed that lncRNA ENST00000453774.1 (lncRNA 74.1) could competitively bind to miR-324-3p, which negatively regulated NRG1 expression. Finally, we also showed that lncRNA 74.1 could control PI3K/AKT signaling pathway mediated by NRG1 to induce cellular autophagy and reduce the fibrogenesis of renal cells. Therefore, this novel regulatory axis of renal fibrosis presents a new potential target to treat renal fibrosis.

## Materials and Methods

### Ethics Statement

The patient sample collection in the current study was conducted under the approval of the Xiangya Hospital, Central South University, in strict adherence to the Guidelines of the *Helsinki Declaration*. Singed informed consents were obtained from each participant prior to specimen collection. The animal experiments were conducted according to the guidelines of National Institutes of Health (NIH) and approved by the Animal Care and Use Committee at the Xiangya Hospital, Central South University. Extensive efforts were made to minimize the suffering of the animals.

### Study Subjects

First, 30 renal biopsy specimens were collected from patients at the Xiangya Hospital, Central South University from February 2016 to December 2016. The obtained specimens were subsequently evaluated for numerous histopathologic features, including renal parenchymal atrophy, glomerulosclerosis, hyalinization, tubular atrophy, interstitial fibrosis, and chronic inflammatory cell infiltration. Accordingly, the specimens were classified as 6 normal specimens, 11 mild fibrosis specimens, and 13 moderate fibrosis specimens. Representative HE staining results are shown in Supplementary Figure 1.

### Cell Treatment

Human renal tubular epithelial cells (HK-2) and HEK293T cells were purchased from the American Type Culture Collection (ATCC, United States). The HK-2 cells were subsequently cultured in Dulbecco’s modified Eagle’s medium/Ham’s F12 (DMEM/F12) (11320082, Gibco, ThermoFisher Scientific, Waltham, MA, United States) containing 10% fetal bovine serum (FBS) (10099141, Gibco). Meanwhile, the HEK293T cells were cultured in high glucose DMEM (10569044, Gibco) supplemented with 10% FBS. All the cells were cultured at 37°C with 5% CO_2_ in air until 70–80% cell confluence was attained, followed by cell passage.

In order to induce renal fibrosis in the HK2 cells (Hu et al., 2016; Xiao et al., 2019; Jung et al., 2020), the cells were treated with TGF-β1 at final concentration of 5 ng/mL (C-63499, PromoCell, Heidelberg, Germany) dissolved in 10 mM of citric acid (pH 3.0). Simultaneously, another set of HK2 cells were treated with an identical volume of 10 mM citric acid (pH 3.0) for 72 h to serve as the control. Next, the initial HK2 cells with TGF-β1 induction were seeded in six-well plates (5 × 10^5^), and further treated with miR-324-3p mimic, miR-324-3p inhibitor, AKT activator SC79 (4 μg/mL; HY-18749; MedChemExpress, United States) dissolved in dimethylsulfoxide (DMSO) (D2650; Sigma-Aldrich, St. Louis, MO, United States), lentivirus delivering lncRNA 74.1 overexpression plasmid (oe-lncRNA 74.1), NRG1 overexpression plasmid (oe-NRG1), short hairpin RNA (shRNA) against lncRNA 74.1 (sh-lncRNA 74.1-1 and sh-lncRNA 74.1-2), or shRNA against NRG1 (sh-NRG1-1 and sh-NRG1-2), or their corresponding negative controls (NCs) (mimic-NC, inhibitor-NC, oe-NC, sh-NC, and DMSO). In addition, 3-MA (2 mM, M9281; Sigma-Aldrich) and rapamycin (100 nm, PZ0329, Sigma-Aldrich) were supplemented to decrease and enhance the autophagy levels, respectively.

A lentivirus delivering lncRNA 74.1 overexpression (oe-lncRNA 74.1) was used to infect cells as previously described (Xiao et al., 2019). In brief, the cDNA sequence of lncRNA 74.1 was directly-synthetized and cloned into the pUC57 clone vector. After being confirmed by sequencing, the fragment was double-digested with *Nhe*I/*Xho*I, and then cloned into the L103 vector (General Biosystems, Changsha, Hunan, China). And the other lentivirus, mimic, and inhibitor were purchased from Sangon Biotech (Shanghai, China). After 24 h, puromycin (P8833, Sigma-Aldrich) was added for cell treatment for a week to obtain stably expressed and interfered cell lines.

### HE Staining

The wax-embedded kidney tissue block was sliced with a vibrotome, flattened in warm water, transferred onto glass slides, dried, and then dewaxed for staining with hematoxylin. After differentiation with hydrochloric acid ethanol, the sections were dehydrated with gradient ethanol and finally sealed with neutral gum for observation under light microscope.

### Bioinformatics Prediction

The miRanda software (v3.3a) was employed to predict the downstream miRNAs of lncRNA 74.1, and the miRNAs presenting with a total score higher than 160 were selected. In addition, the Starbase database was adopted to predict the downstream target genes of lncRNA 74.1. The two prediction results were intersected to obtain the target gene of lncRNA 74.1. Additionally, the miRDB, mirDI, and miRecords databases were adopted to predict the target genes of miR-324-3p, and the intersection was selected as the candidate target gene.

Finally, the renal fibrosis model-related expression dataset GSE100631 was retrieved from the GEO database, and differential analysis was conducted using the R software limma package, with | logfFC| > 0.9 and *p* < 0.05 serving as the threshold.

### Immunofluorescence

The HK2 cells were fixed with 4% paraformaldehyde for 15 min, treated with 0.2% Triton X-100 for 20 min at room temperature, and then blocked with 5% goat serum diluted by 0.3% phosphate-buffered saline with Tween (PBST) at room temperature for 1 h. Subsequently, the cells were incubated with the diluted rabbit anti-human LC3B primary antibody (ab51520, dilution ratio of 1: 1,000, Abcam, Cambridge, United Kingdom) at room temperature for 1 h, followed by staining with the fluorescein isothiocyanate (FITC)-labeled goat anti-rabbit secondary antibody (ab6717, dilution ratio of 1: 500, Abcam). Finally, the results were observed under a microscope (BX63, Olympus, Tokyo, Japan).

### RNA Isolation and Quantification

Total RNA content was extracted from the tissues and cells using the TRIzol agent (16096020, Thermo Fisher Scientific). The cDNA of miRNA was synthesized using the miRNA First Strand cDNA Synthesis kit (Tailing Reaction) (B532451, Sangon Biotech), while the mRNA was obtained using the BeyoRT^TM^ II First Strand cDNA Synthesis kit (RNase H-) (D7168L, Beyotime, Shanghai, China). Subsequently, RT-qPCR was performed using the AceQ^®^ Universal SYBR qPCR Master Mix kit (Q511-02, Vazyme Biotech, Nanjing, China) on a CFX96^TM^ Real-Time PCR Detection System (Bio-Rad Laboratories, Hercules, CA, United States). U6 was regarded as the internal control for miR-324-3p, while glyceraldehyde-3-phosphate dehydrogenase (GAPDH) served as the internal control for lncRNA 74.1 and NRG1. The primer of U6 was obtained using the microRNA reverse transcription kit, and the primer sequences of lncRNA 74.1, miR-324-3p, NRG1, and GAPDH were provided by Sangon Biotech (Supplementary Table 1). Fold changes were calculated based on the 2^–ΔΔ^ ^Ct^ method.

### Protein Isolation and Quantification

Total protein content was isolated from the tissues and cells using radio immunoprecipitation assay (RIPA) lysis buffer (P0013B, Beyotime), from which the nuclear and cytoplasmic proteins were isolated using the related kit (P0028, Beyotime) (Yang et al., 2010; Gu et al., 2019). The protein concentration was then quantified with a bicinchoninic acid (BCA) kit (23229, Thermo Fisher Scientific). Subsequently, protein samples were transferred onto a PVDF membrane (1620177, Bio-Rad Laboratories), which were then blocked with 5% skim milk or 5% bovine serum albumin (BSA) for 1 h, followed by immunoblotting. The included primary antibodies were as follows: GAPDH (ab181602, dilution ratio of 1: 5,000, Abcam), NRG1 (ab53104, dilution ratio of 1: 1,000, Abcam), PI3K (4292, dilution ratio of 1: 1,000, Cell Signaling Technology, Danvers, MA, United States), p-PI3K (PA5-17387, dilution ratio of 1: 1,000, Thermo Fisher Scientific), Akt (9272, dilution ratio of 1: 1,000, Cell Signaling Technology), p-Akt (4058, dilution ratio of 1: 1,000, Cell Signaling Technology), LC3B (ab51520, dilution ratio of 1: 1,000, Abcam), ATG5 (ab108327, dilution ratio of 1: 1,000, Abcam), ATG7 (ab52472, dilution ratio of 1: 1,000, Abcam), P62 (ab109012, dilution ratio of 1: 1,000, Abcam), β-actin (ab8227, 1: 1,000, Abcam), Fibronectin (ab32419, dilution ratio of 1: 1,000, Abcam), Collagen I (ab34710, dilution ratio of 1: 1,000, Abcam), and α-SMA (ab32575, dilution ratio of 1: 1,000, Abcam). The secondary antibody used in this experiment was the horseradish peroxidase (HRP)-labeled goat anti-rabbit immunoglobulin G (IgG; ab6721, dilution ratio of 1: 5,000, Abcam). The Image Quant LAS 4000C software (GE Healthcare, Madison, WI, United States) was then adopted for result visualization, and the protein content was quantitatively evaluated as the ratio of the gray value of each protein to that of the internal reference.

### Dual-Luciferase Reporter Gene Assay

The miRanda software (v3.3a) was adopted to predict the binding site between lncRNA 74.1 and miR-324-3p, while the binding site between miR-324-3p and NRG1 was predicted using an online database. The artificially-synthetized 3′UTR of lncRNA 74.1 and the miR-324-3p gene fragments were inserted into the pGL3-basic reporter (E1751, Promega, Madison, WI, United States). The complementary sequence mutation sites of the seed sequence were designed on the wild-type (WT) lncRNA 74.1 and miR-324-3p (lncRNA 74.1 Wt and NRG1 Wt). The target fragment was subsequently inserted into the pGL3-basic reporter plasmids by restriction endonuclease digestion using the T4 DNA ligase (M0204S, New England Biolabs, Beverly, MA, United States). After confirming the sequences, the Wt plasmids and mutant (Mut) plasmids (lncRNA 74.1 mut and NRG1 mut) were also co-delivered using the miR-324-3p mimic or mimic-NC into the HEK-293T cells. Luminescence was then detected using a Dual-Luciferase^®^ Reporter Assay System (E1910, Promega). A comparison of the luminescent signal reflecting the activation of the target reporter gene was conducted based on the ratio of the Firefly luciferase relative light units (RLU) to the Renilla luciferase RLU value. All reporter plasmids were constructed by Sangon Biotech.

### RNA Immunoprecipitation

The binding of lncRNA 74.1 and miR-324-3p with the AGO2 protein was detected using RNA Immunoprecipitation (RIP) kits (17-701, Millipore, Bedford, MA, United States) according to the manufacturer’s instructions. Cells were prepared in RIPA lysate, and a portion served as input. The remaining lysate was incubated with 50 μL beads. Next, the RNA-protein complexes were immunoprecipitated using anti-Ago2 (dilution ratio of 1: 100, ab32381, Abcam) or the normal goat IgG (control) (dilution ratio of 1: 100, ab200699, Abcam). The obtained RNAs (lncRNA 74.1 and miR-324-3p) were purified and subjected to RT-qPCR analysis. The RIP RNA fraction Ct value was normalized to the input RNA fraction Ct value.

### Fluorescence *in situ* Hybridization Assay

The subcellular localization of lncRNA 74.1 was measured using a fluorescence *in situ* hybridization (FISH) kit (F32956, Thermo Fisher Scientific). Next, the Digoxigenin-labeled RNA probe of lncRNA 74.1 (5′–3′: AAGGGGGACCTAGGTCCTGGGGGAGGTC) was hybridized with the metaphase chromosomes of the cells. The metaphase chromosomes were then counterstained with 4′,6-diamidino-2-phenylindole (DAPI) (D9542, Sigma-Aldrich) for 10 min after the hybridization process. The results were observed under a confocal laser scanning microscope (FV1000, Olympus).

### Transmission Electron Microscope

The HK-2 cells (density of 1 × 10^5^) were fixed with 2.5% glutaraldehyde at 4°C for 4 h, and then with 1% osmium tetroxide for 1 h. Following dehydration, the cells were sliced and stained with uranyl acetate and lead citrate. After observation under Tecnai 10 transmission electron microscope (TEM) (FEI Company, Hillsboro, OR, United States), the number of autophagosomes was calculated in a minimum of 6 cells.

### Unilateral Ureteral Obstruction Animal Model

A unilateral ureteral obstruction (UUO) model was established in male C57BL/6J mice (aged 4 months). The mice were grouped into sham, UUO + LV-NC, and UUO + LV-74.1 groups (four mice in each group). Ureteral obstruction was performed by ligation of the left ureter using a 3-0 silk *via* a left lateral incision. The sham operated mice were regarded as controls. The sham operated mouse renal specimens were harvested at day 14. The UUO + LV-NC and UUO + LV-74.1 mice were then injected with 2 × 10^7^ TU LV-NC and 2 × 10^7^ TU LV-74.1 respectively *via* the tail vein.

### Lentivirus-Mediated lncRNA 74.1 Overexpression *in vitro* and *in vivo*

To obtain lncRNA 74.1 gain-of-function models *in vitro* and *in vivo*, lentivirus-mediating overexpression was performed using the lentivirus recombinant overexpression plasmid construction and package. The cDNA sequence of lncRNA 74.1 was directly-synthetized and cloned into the pUC57 clone vector. After sequencing, the fragment was double-digested with Nhel/Xhol and then cloned into the L103 vector (General Biosystems). The lentivirus package was prepared by Auragene biotechnology (Changsha, China). HK-2 cells were infected with Lv-74.1 or Lv-Con (blank L103 vector) at the MOI value of 10. Mice were injected with either 2 × 10^7^ TU LV-74.1 or Lv-con *via* the tail vein.

### Statistical Analysis

Statistical analyses were performed using the SPSS 21.0 statistical software (IBM Corp. Armonk, NY, United States) and GraphPad v8.4.2.679 software. Measurement data were expressed as mean ± standard deviation. Data between two groups were compared using the unpaired *t-*test (unpaired data). One-way analysis of variance (ANOVA) was adopted for data comparisons among multiple groups, followed by Tukey’s *post hoc* test with corrections for multiple comparisons. A value of *p* < 0.05 was indicative of statistical significance.

## Results

### LncRNA 74.1 Binds to miR-324-3p to Promote Autophagy and Inhibit Renal Fibrosis

Our previous literature results illustrated that lncRNA 74.1 is poorly expressed in clinical tissue samples and in the TGF-β1-treated HK-2 cell fibrosis model, while treatment with lncRNA 74.1 can alleviate renal fibrosis through an autophagy-mediated oxidative stress defense mechanism (Xiao et al., 2019). First, to determine whether lncRNA74.1 modulated autophagy in renal fibrosis, we altered autophagy levels directly by treatment of the cells with 3-MA and rapamycin, respectively and then performed Western blot analysis to detect the expression of LC3-II/I. The results showed that (1) the relative expression of LC3-II/I was enhanced significantly in the oe-lncRNA74.1 group compared with the oe-NC, (2) when the autophagy inhibitor 3-MA was added, the LC3-II/I expression decreased obviously in the oe-lncRNA74.1 group compared with the oe-lncRNA74.1 group, and (3) when the autophagy agonist rapamycin was added, the LC3-II/I expression further increased in the oe-Lv-74.1 group compared with the oe-lncRNA74.1 group (Supplementary Figure 2).

**FIGURE 1:**
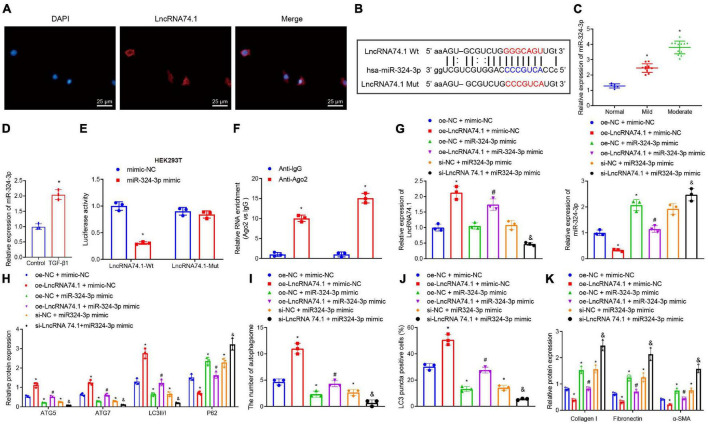
LncRNA 74.1 downregulates miR-324-3p to promote cell autophagy and inhibit renal fibrosis. **(A)** The subcellular localization of lncRNA 74.1 by FISH assay (×400); **(B)** prediction of binding site between lncRNA 74.1 and miR-324-3p by miRanda software (v3.3a); **(C)** RT-qPCR results of miR-324-3p expression in 30 renal tissue samples; **(D)** RT-qPCR results of miR-324-3p expression in HK-2 cells exposed to TGF-β1; **(E)** targeting relationship between lncRNA 74.1 and miR-324-3p by dual-luciferase reporter gene assay; **(F)** binding of lncRNA 74.1 and miR-324-3p with Ago2 by RIP assay; **(G)** RT-qPCR results of lncRNA 74.1 and miR-324-3p expression in HK-2 cells with different treatments; **(H)** level of autophagy-related proteins by Western blot; **(I)** TEM observation of autophagosomes in HK-2 cells with different treatments; **(J)** proportion of LC3 positive cells in the HK-2 cells with different treatments by immunofluorescence assay; **(K)** protein level of fibrosis markers in HK-2 cells with different treatments by Western blot. *p < 0.05 as compared with normal, control, mimic-NC, anti-lgG, or oe-NC + mimic-NC; ^#^*p* < 0.05 as compared with oe-LncRNA74.1 + mimic-NC; ^&^*p* < 0.05 as compared with si-NC + miR-324-3p mimic. The measurement data were expressed as mean ± standard deviation. Data between the two groups were compared using the unpaired *t-*test. One-way ANOVA was used for data comparisons among multiple groups, followed by Tukey’s *post hoc* test. The cell experiments were repeated three times independently.

To further investigate the mechanism of lncRNA 74.1 in regulating autophagy, FISH assay was performed to detect the subcellular localization of lncRNA 74.1. This revealed that lncRNA 74.1 was predominantly localized in the cytoplasm, as distinct from DAPI staining of the nucleus (Figure 1A). Since lncRNA is located in the cytoplasm and is usually closely related to its function as a ceRNA (Zhang et al., 2018), we speculated that lncRNA 74.1 may play a regulatory role through ceRNA mechanism. Subsequently, the miRanda software was adopted to identify the miRNAs that could bind to lncRNA 74.1(Supplementary Table 2). In addition, the Starbase database was retrieved to predict the miRNA regulated by lncRNA 74.1 (Supplementary Table 3), and then combined with the prediction results of miRanda software to plot a Venn map (Supplementary Figure 3). We thus found that miR-324-3p was the only miRNA at the intersection and that miR-324-3p could apparently be regulated by lncRNA 74.1. Also, as previously reported, miR-324-3p was highly expressed in renal fibrosis, and further promoted the incidence and development of renal fibrosis (Macconi et al., 2012). Therefore, miR-324-3p was selected for subsequent experimentation in the current study. As shown in Figure 1B, the miRanda software was employed to predict the presence of a binding site between lncRNA 74.1 and miR-324-3p, and the lncRNA 74.1 mut sequence was designed according to the prediction results. RT-qPCR was then performed to detect the miR-324-3p expression patterns in the tissues and cells, which demonstrated that miR-324-3p expression was augmented upon aggravation of renal fibrosis, suggesting that miR-324-3p may play an important role in the process of renal fibrosis (Figure 1C). In comparison with the control cells, cells with the TGF-β1 treatment presented with elevated miR-324-3p expression (Figure 1D). Next, RT-qPCR was performed to detect the transfection efficacy of miR-324-3p mimic or inhibitor in the HEK293T and TGF-β treated HK-2 cells, which revealed that cells transfected with miR-324-3p mimic exhibited significantly increased miR-324-3p expression relative to those transfected with the mimic-NC. On the other hand, cells transfected with the miR-324-3p inhibitor presented with significantly decreased miR-324-3p expression compared with those transfected with inhibitor-NC (Supplementary Figure 4A). Meanwhile, dual-luciferase reporter gene assay results (Figure 1E) revealed that, in comparison with mimic-NC treatment, miR-324-3p mimic significantly decreased the fluorescence signal of lncRNA74.1-Wt, while no significant difference was observed in lncRNA74.1-Mut signal. Moreover, the RIP assay showed that anti-Ago2 promoted the expressions of lncRNA 74.1 and miR-324-3p compared to anti-IgG (*p* < 0.05, Figure 1F). RT-qPCR also revealed the successful transfection of lncRNA 74.1 overexpression plasmids in the cells. In addition, we found that augmenting the lncRNA 74.1 expression brought about a decreased miR-324-3p expression (*p* < 0.05, Supplementary Figure 4B). The aforementioned data demonstrated that lncRNA 74.1 could bind to miR-324-3p and negatively regulate its expression.

Additionally, we explored the effect of lncRNA 74.1 and miR-324-3p on cellular autophagy and renal fibrosis. RT-qPCR illustrates that, in comparison to the cells treated with oe-NC + mimic-NC, those treated with oe-lncRNA 74.1 + mimic-NC exhibited high lncRNA 74.1 expression and low miR-324-3p expression, while cells treated with oe-NC + miR-324-3p mimic and si-NC + miR-324-3p mimic showed enhanced miR-324-3p expression, with no significant change in lncRNA 74.1 expression. Compared with cells treated with oe-lncRNA 74.1 + mimic-NC, those treated with oe-lncRNA 74.1 + miR-324-3p mimic presented increased miR-324-3p expression. Compared with cells treated with si-NC + miR-324-3p mimic, si-lncRNA74.1 + miR-324-3p mimic treatment brought about reduced lncRNA 74.1 expression and enhanced miR-324-3p expression (Figure 1G). miR-324-3p expression was significantly increased after lncRNA74.1 was silenced alone in cells (Supplementary Figure 5).

Furthermore, the levels of autophagy-related proteins were detected by Western blot analysis (Figure 1H). We found that, compared to the HK-2 cells treated with oe-NC + mimic-NC, the cells treated with oe-lncRNA 74.1 + mimic-NC presented with significantly increased protein levels of ATG5, ATG7, and LC3II/I, and notably decreased P62 protein level, while opposite results were documented in the cells treated with oe-NC + miR-324-3p mimic. Compared to the HK-2 cells treated with oe-lncRNA 74.1 + mimic-NC, oe-lncRNA 74.1 + miR-324-3p mimic treatment brought about reduced protein levels of ATG5, ATG7, and LC3II/I, but enhanced the P62 protein level. Compared with si-NC + miR-324-3p mimic treatment, si-lncRNA74.1 + miR-324-3p mimic treatment precipitated reduced protein levels of ATG5, ATG7, and LC3II/I, but enhanced the P62 protein level.

Figure 1I and Supplementary Figure 6A illustrate the results of TEM observation, demonstrating that treatment with oe-lncRNA 74.1 + mimic-NC promoted the number of autophagosomes, while oe-NC + miR-324-3p mimic reduced the number of autophagosomes. Compared with oe-lncRNA 74.1 + mimic-NC treatment, oe-lncRNA 74.1 + miR-324-3p mimic brought about a reduced number of autophagosomes, as did thesi-lncRNA74.1 + miR-324-3p mimic treatment compared with the si-NC + miR-324-3p mimic treatment. Meanwhile, immunofluorescence assays revealed an increased proportion of LC3 positive cells in the HK-2 cells following treatment with oe-lncRNA 74.1 + mimic-NC, and decreased proportion of LC3 positive cells in those treated with oe-NC + miR-324-3p mimic. Compared with oe-lncRNA 74.1 + mimic-NC treatment, the oe-lncRNA 74.1 + miR-324-3p mimic treatment decreased the proportion of LC3 positive cells as did the si-lncRNA74.1 + miR-324-3p mimic treatment compared with the si-NC + miR-324-3p mimic treatment (Figure 1J and Supplementary Figure 7A). In addition, Western blot was performed to measure protein levels of various fibrosis markers (Collagen I, Fibronectin, and α-SMA). As shown in Figure 1K and Supplementary Figure 8A, Collagen I, Fibronectin, and α-SMA protein levels were decreased in the HK-2 cells treated with oe-lncRNA 74.1 + mimic-NC, but opposite results were noted in cells treated with oe-NC + miR-324-3p mimic. Compared with the oe-lncRNA 74.1 + mimic-NC treatment, the oe-lncRNA 74.1 + miR-324-3p mimic brought about increased protein levels of the fibrosis markers, as did the treatment with si-lncRNA74.1 + miR-324-3p mimic compared with the si-NC + miR-324-3p mimic treatment. Altogether, these results suggested that lncRNA 74.1 promoted cell autophagy and inhibited renal fibrosis by down-regulating miR-324-3p.

### miR-324-3p Targets and Inhibits NRG1 Expression

**FIGURE 2:**
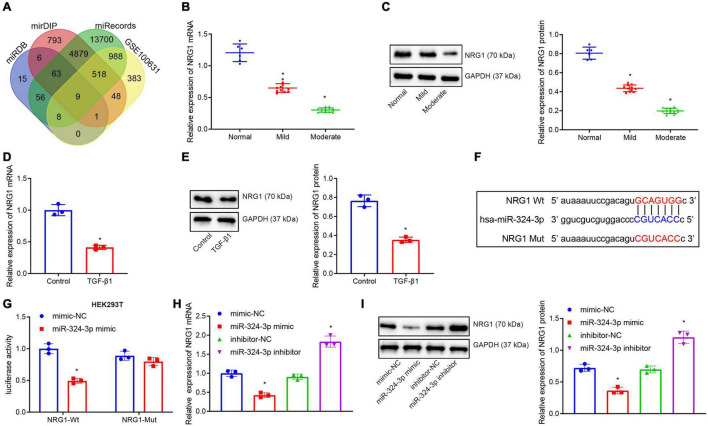
miR-324-3p targets and negatively regulates NRG1. **(A)** Venn diagram of the target genes of miR-324-3p in miRDB, mirDIP, and miRecords databases; **(B)** RT-qPCR results of NRG1 mRNA expression in 30 renal tissue samples; **(C)** Western blot results of NRG1 protein expression in 30 renal tissue samples; **(D)** RT-qPCR results of NRG1 mRNA expression in HK-2 cells exposed to TGF-β1; **(E)** Western blot results of NRG1 protein expression in HK-2 cells exposed to TGF-β1; **(F)** online prediction of the binding site of miR-324-3p and NRG1 mRNA 3′UTR; **(G)** targeting relationship between miR-324-3p and NRG1 by dual-luciferase reporter gene assay; **(H)** RT-qPCR results of the NRG1 mRNA expression after miR-324-3p overexpression or silencing; **(I)** Western blot results of the NRG1 protein expression after miR-324-3p overexpression or silencing. *p < 0.05 as compared with normal, control, mimic-NC, or inhibitor-NC. The measurement data were expressed as mean ± standard deviation. Data between the two groups were compared using the unpaired *t-*test. One-way ANOVA was used for data comparisons among multiple groups, followed by Tukey’s *post hoc* test. The cell experiments were repeated three times independently.

Subsequently, the miRDB, mirDIP, and miRecords databases were retrieved to predict the target gene sets of miR-324-3p, and the expression dataset GSE100631 of renal fibrosis samples was obtained from the GEO database, whereupon differential analysis was performed using the R software limma package to obtain the down-regulated genes. Nine candidate genes were found in the intersection of the predicted gene sets and the down-regulated gene sets (Figure 2A). An existing study reported that NRG1 exerts an inhibitory role in several fibrosis diseases including renal fibrosis (Shakeri et al., 2021). Thus, thus NRG1 was selected as our study subject. Next, RT-qPCR and Western blot assays revealed that NRG1 expression was diminished upon aggravation of renal fibrosis (Figures 2B,C). In addition, decreased NRG1 expression was found in the HK-2 cells exposed to TGF-β1 (Figures 2D,E). Figure 2F displays prediction of the binding site between miR-324-3p and NRG1 mRNA 3′UTR, which was further confirmed by dual-luciferase reporter gene assay (Figure 2G). In contrast to the mimic-NC transfection, the co-transfection of miR-324-3p mimic and NRG1-Wt decreased the luminescent signal in HEK293T cells, whereas no significant difference in luminescent signal was noted following co-transfection with miR-324-3p mimic and NRG1-Mut. RT-qPCR and Western blot analysis showed that miR-324-3p overexpression brought about decreased expression of NRG1, while miR-324-3p down-regulation resulted in increased NRG1 expression (Figures 2H,I). These findings highlighted that miR-324-3p could target NRG1 and negatively regulate its expression.

### NRG1 Overexpression Reverses miR-324-3p-Inhibited Autophagy to Alleviate Renal Fibrosis

**FIGURE 3:**
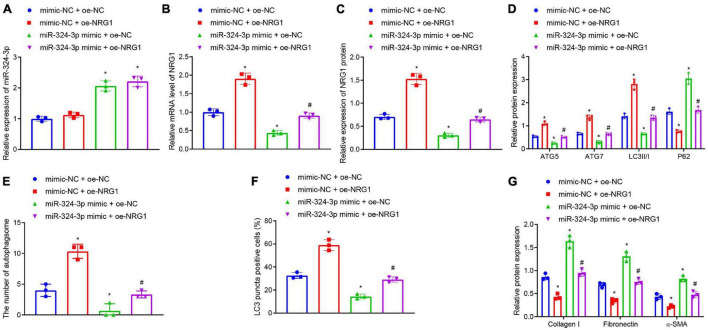
NRG1 overexpression promotes the miR-324-3p-inhibited autophagy to alleviate renal fibrosis. **(A)** RT-qPCR results of miR-324-3p expression in HK-2 cells with different treatments; **(B)** RT-qPCR results of NRG1 mRNA expression in HK-2 cells with different treatments; **(C)** Western blot results of NRG1 protein expression in HK-2 cells with different treatments; **(D)** level of autophagy-related proteins by Western blot; **(E)** TEM observation of autophagosomes in HK-2 cells with different treatments; **(F)** proportion of LC3 positive cells in the HK-2 cells with different treatments by immunofluorescence assay; **(G)** protein level of fibrosis markers in HK-2 cells with different treatments by Western blot; *p < 0.05 as compared with oe-NC + mimic-NC; ^#^*p* < 0.05 as compared with miR-324-3p mimic + oe-NC. The measurement data were expressed as mean ± standard deviation. One-way ANOVA was used for data comparisons among multiple groups, followed by Tukey’s *post hoc* test. The cell experiments were repeated three times independently.

Furthermore, we tested whether miR-324-3p inhibited autophagy and promoted renal fibrosis by inhibiting NRG1 expression. RT-qPCR (Figures 3A–C and Supplementary Figure 8B) depicted that, compared with the mimic-NC + oe-NC treatment, miR-324-3p mimic + oe-NC treatment enhanced miR-324-3p expression and reduced NRG1 expression, while mimic-NC + oe-NRG1 treatment only led to enhanced NRG1 expression. Compared with miR-324-3p mimic + oe-NC treatment, miR-324-3p mimic + oe-NRG1 treatment resulted in enhanced NRG1 expression. In addition, Western blot analysis (Figure 3D) revealed that, relative to the cells treated with oe-NC + mimic-NC, the cells treated with the oe-NC + miR-324-3p mimic showed significantly decreased protein levels of ATG5, ATG7, and LC3II/I and notably increased P62 protein level, while opposite results were recorded in the cells treated with mimic-NC + oe-NRG1. In comparison to the cells treated with oe-NC + miR-324-3p mimic, those treated with miR-324-3p mimic + oe-NRG1 exhibited increased protein levels of ATG5, ATG7, and LC3II/I, and reduced P62 protein level. Additionally, TEM observation results (Figure 3E and Supplementary Figure 6B) demonstrated that miR-324-3p mimic reduced the number of autophagosomes, while NRG1 overexpression restored the autophagosomes reduced by miR-324-3p mimic. Relative to cells treated with oe-NC + miR-324-3p mimic, those treated with miR-324-3p mimic + oe-NRG1 exhibited an increased number of autophagosomes. As depicted by immunofluorescence assay, a decreased proportion of LC3 positive cells were observed among the HK-2 cells treated with oe-NC + miR-324-3p mimic, while the opposite held for the HK-2 cells following mimic-NC + oe-NRG1 or miR-324-3p mimic + oe-NRG1 treatment (Figure 3F and Supplementary Figure 7B). As shown in Figure 3G, the protein levels of Collagen I, Fibronectin, and α-SMA were elevated after miR-324-3p overexpression, but were decreased following NRG1 overexpression. Meanwhile, compared to the cells treated with oe-NC + miR-324-3p mimic, those treated with miR-324-3p mimic + oe-NRG1 exhibited reduced protein levels of Collagen I, Fibronectin, and α-SMA. The aforementioned results suggested that NRG1 overexpression could improve renal fibrosis by annulling the inhibitory effect of miR-324-3p on autophagy.

### LncRNA 74.1 Antagonizes the Inhibitory Effect of miR-324-3p on the Expression of NRG1

**FIGURE 4:**
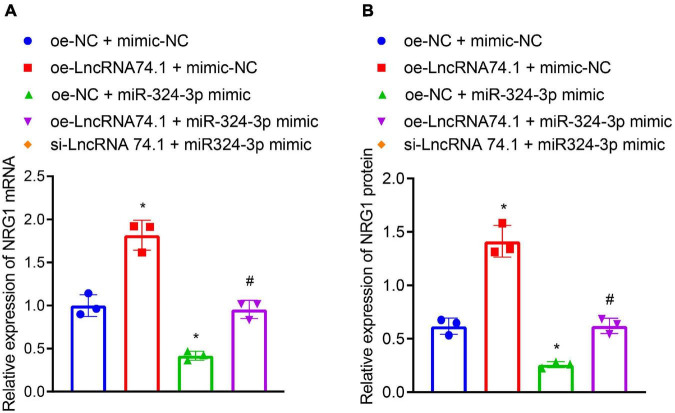
LncRNA 74.1 binds to miR-324-3p to promote NRG1 expression. **(A)** RT-qPCR results of NRG1 mRNA expression in HK-2 cells with different treatments; **(B)** Western blot results of NRG1 protein expression in HK-2 cells with different treatments; *p < 0.05 as compared with oe-NC + mimic-NC; ^#^*p* < 0.05 as compared with oe-lncRNA 74.1 + mimic-NC. The measurement data were expressed as mean ± standard deviation. One-way ANOVA was used for data comparisons among multiple groups, followed by Tukey’s *post hoc* test. The cell experiments were repeated three times independently.

We further verified that lncRNA 74.1 promoted NRG1 expression by competitively binding to miR-324-3p. RT-qPCR and Western blot analysis were performed to detect expression patterns of NRG1. As displayed in Figures 4A,B, compared to the HK-2 cells with oe-NC + mimic-NC treatment, those treated with oe-lncRNA 74.1 + mimic-NC exhibited increased NRG1 expression, while those that had undergone oe-NC + miR-324-3p mimic treatment had decreased NRG1 expression; compared with the oe-lncRNA 74.1 + mimic-NC treatment, cells treated with oe-lncRNA 74.1 + miR-324-3p mimic presented with decreased NRG1 expression. These results highlighted that lncRNA 74.1 promoted NRG1 expression, miR-324-3p inhibited NRG1 expression, and lncRNA 74.1 antagonized the inhibitory effect of miR-324-3p on NRG1.

### NRG1 Silencing Inhibits the lncRNA 74.1-Induced Autophagy to Promote Renal Fibrosis

**FIGURE 5:**
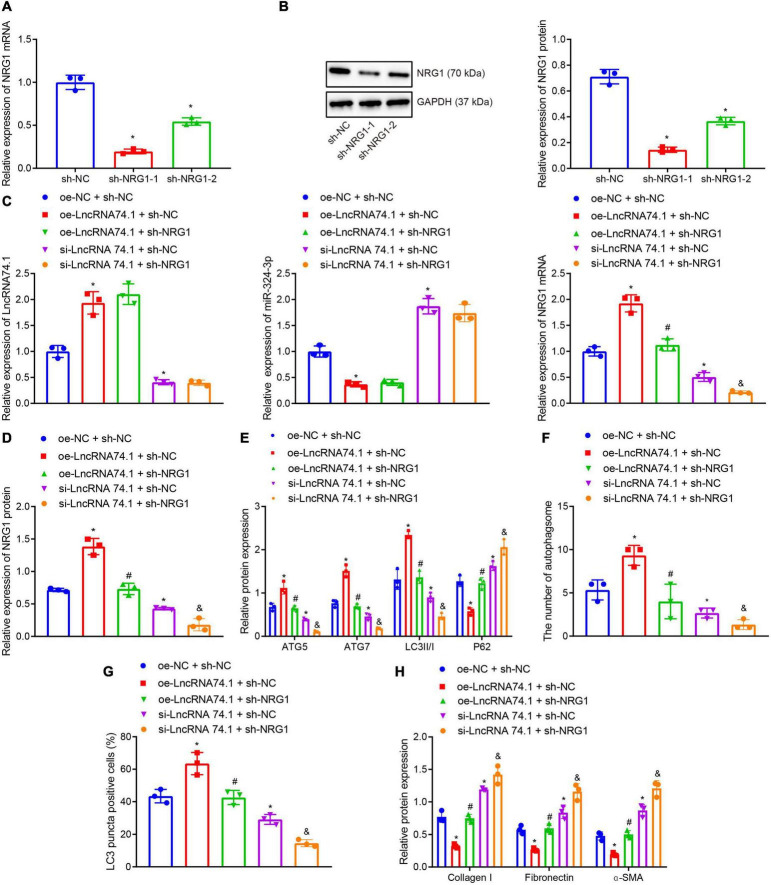
NRG1 silencing inhibits the lncRNA 74.1-induced autophagy to aggravate renal fibrosis. **(A)** The efficacy of NRG1 silencing by RT-qPCR; **(B)** the efficacy of NRG1 silencing by Western blot; **(C)** RT-qPCR results of the expressions of lncRNA 74.1, miR-324-3p, and NRG1 mRNA in HK-2 cells with different treatments; **(D)** Western blot results of NRG1 protein expression in HK-2 cells with different treatments; **(E)** level of autophagy-related proteins by Western blot; **(F)** TEM observation of autophagosomes in HK-2 cells with different treatments; **(G)** proportion of LC3 positive cells in the HK-2 cells with different treatments by immunofluorescence assay; **(H)** the protein levels of fibrosis markers in HK-2 cells with different treatments by Western blot; *p < 0.05 as compared with oe-NC + sh-NC; ^#^*p* < 0.05 as compared with oe-lncRNA 74.1 + sh-NC, ^&^*p* < 0.05 as compared with si-LncRNA74.1 + sh-NC. The measurement data were expressed as mean ± standard deviation. One-way ANOVA was used for data comparisons among multiple groups, followed by Tukey’s *post hoc* test. The cell experiments were repeated three times independently.

Furthermore, we verified that lncRNA 74.1 promoted autophagy and inhibited renal fibrosis through NRG1. RT-qPCR and Western blot analysis were performed to detect the efficacy of NRG1 silencing in HK-2 cells (Figures 5A,B), which revealed that both sh-NRG1-1 and sh-NRG1-2 brought about remarkable decreases in NRG1 expression. Due to superior silencing efficacy of sh-NRG1-1, we selected it for subsequent experimentation. Figure 5C illustrates the expression of lncRNA 74.1, miR-324-3p, and NRG1 as detected by RT-qPCR, and Figure 5D displays the NRG1 protein expression measured with Western blot analysis. The results revealed that, in comparison to the cells treated with oe-NC + sh-NC, those treated with oe-lncRNA 74.1 + sh-NC showed increased lncRNA 74.1 and NRG1 expression but reduced miR-324-3p expression, while cells treated with si-lncRNA 74.1 + sh-NC had reduced lncRNA 74.1 and NRG1 expression but enhanced miR-324-3p expression. Meanwhile, compared to cells treated with oe-lncRNA 74.1 + sh-NC, those treated with oe-lncRNA 74.1 + sh-NRG1 exhibited reduced NRG1 expression. Compared with si-lncRNA 74.1 + sh-NC, si-lncRNA74.1 + sh-NRG1 exhibited reduced NRG1 expression.

Moreover, Western blot analysis (Figure 5E) showed that, in comparison to the cells treated with oe-NC + sh-NC, the protein levels of ATG5, ATG7, and LC3II/I were obviously increased and the P62 protein level was notably decreased in cells treated with oe-lncRNA 74.1 + sh-NC; opposite findings were seen in cells treated with si-lncRNA 74.1 + sh-NC. Meanwhile, reduced protein levels of ATG5, ATG7, and LC3II/I, and increased P62 protein level were observed in the cells treated with oe-lncRNA 74.1 + sh-NRG1 compared to the oe-lncRNA 74.1 + sh-NC treatment, and similar trends were observed in cells treated with si-lncRNA74.1 + sh-NRG1 compared with si-lncRNA 74.1 + sh-NC. As illustrated by TEM observation results (Figure 5F), lncRNA 74.1 overexpression elevated the number of autophagosomes, whereas further NRG1 silencing inhibited the autophagosomes promoted by overexpression of lncRNA 74.1; meanwhile, silencing lncRNA 74.1 reduced the number of autophagosomes, whereas further silencing NRG1 lead to still more significant reduction in number of autophagosomes. Figure 5G depicts the immunofluorescence assay results, which revealed that the proportion of LC3 positive cells in the HK-2 cells was elevated in response to lncRNA 74.1 overexpression, which could be reduced after further NRG1 silencing. Also, lncRNA 74.1 silencing evoked a reduced proportion of LC3 positive cells among the HK-2 cells, and a still more obvious reduction was observed after further silence of NRG1. In addition, Western blot (Figure 5H) revealed that the protein levels of Collagen I, Fibronectin, and α-SMA were reduced after lncRNA 74.1 overexpression, while opposite effects were seen after lncRNA 74.1 silencing. Compared to the cells treated with overexpressed lncRNA 74.1 alone, those treated both with overexpressed lncRNA 74.1 and silenced NRG1 showed enhanced protein levels of Collagen I, Fibronectin, and α-SMA, while compared to the cells treated with silenced lncRNA 74.1, those treated with silenced lncRNA74.1 + silenced NRG1 presented with enhanced protein levels of Collagen I, Fibronectin, and α-SMA. The aforementioned data proved that NRG1 silencing could aggravate renal fibrosis by inhibiting the autophagy induced by lncRNA 74.1.

### LncRNA 74.1 Promotes Autophagy and Inhibits Renal Fibrosis *via* Inactivation of PI3K/AKT Signaling Pathway

**FIGURE 6:**
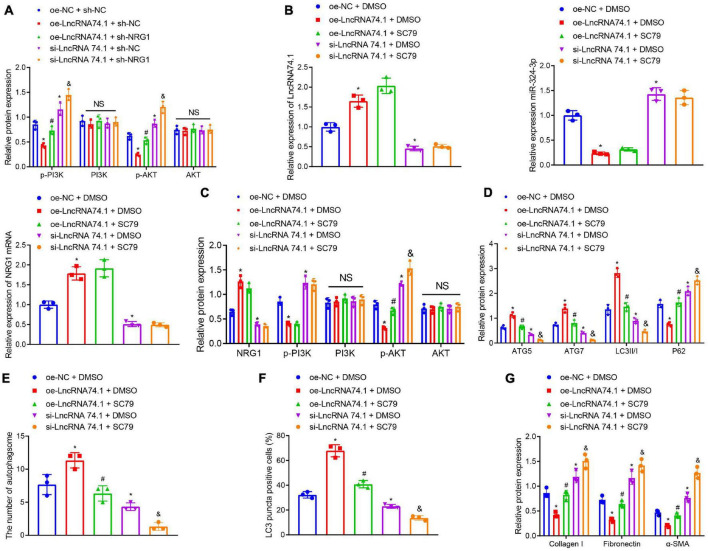
LncRNA 74.1 inactivates the PI3K/AKT signaling pathway to promote autophagy and inhibit renal fibrosis. **(A)** Western blot results of PI3K, p-PI3K, AKT, and p-AKT expressions in HK-2 cells with different treatments; **(B)** RT-qPCR results of the expressions of lncRNA 74.1, miR-324-3p, and NRG1 mRNA in cells with different treatments; **(C)** Western blot results of NRG1, PI3K, p-PI3K, AKT, and p-AKT expressions in HK-2 cells with different treatments; **(D)** level of autophagy-related proteins by Western blot; **(E)** TEM observation of autophagosomes in HK-2 cells with different treatments; **(F)** proportion of LC3 positive cells in the HK-2 cells with different treatments by immunofluorescence assay; **(G)** protein levels of fibrosis markers in HK-2 cells with different treatments by Western blot; *p < 0.05 as compared with oe-NC + sh-NC or oe-NC + DMSO; ^#^*p* < 0.05 as compared with oe-lncRNA 74.1 + sh-NC or oe-lncRNA 74.1 + DMSO; ^&^*p* < 0.05 as compared with si-lncRNA74.1 + sh-NC or si-lncRNA74.1 + DMSO. The measurement data were expressed as mean ± standard deviation. One-way ANOVA was used for data comparisons among multiple groups, followed by Tukey’s *post hoc* test. The cell experiments were repeated three times independently.

Existing data suggests that NRG1 can inhibit the PI3K/AKT signaling pathway, which may suppress cellular autophagy and facilitate several fibrotic diseases including renal fibrosis (Vermeulen et al., 2017; Dou et al., 2019; Wan et al., 2019). Consequently, we speculated about an involvement of the PI3K/AKT signaling pathway in the role of lncRNA 74.1 in autophagy and renal fibrosis. In order to test this prediction, Western blot analysis was performed to determine the protein levels of PI3K, p-PI3K, AKT, and p-AKT. We found that oe-lncRNA 74.1 treatment decreased the levels of p-PI3K and p-AKT, while si-lncRNA74.1 increased levels of p-PI3K and p-AKT. Compared with oe-lncRNA 74.1 treatment, oe-lncRNA74.1 + sh-NRG1 treatment enhanced levels of p-PI3K and p-AKT. Compared with si-lncRNA74.1 + sh-NC, si-lncRNA74.1 + sh-NRG1 treatment led to enhanced levels of p-PI3K and p-AKT. No significant difference in the protein levels of PI3K and AKT among the groups was observed (Figure 6A). Collectively, lncRNA 74.1 could promote NRG1 expression, thereby blocking the PI3K/AKT signaling pathway.

Additionally, we treated HK-2 cells with the AKT activator, SC79, followed by measurement of autophagy and fibrosis. RT-qPCR manifested that, in comparison with the cells treated with oe-NC + DMSO, those treated with oe-lncRNA 74.1 + DMSO showed enhanced lncRNA 74.1 and NRG1 expression, but reduced miR-324-3p expression, while those treated with si-lncRNA 74.1 + DMSO showed opposite results. Compared with oe-lncRNA 74.1 + DMSO, the cells treated with oe-lncRNA 74.1 + SC79 exhibited no change in lncRNA 74.1, NRG1, and miR-324-3p expression. Compared with si-lncRNA 74.1 + DMSO, treatment with si-lncRNA 74.1 + SC79 exhibited no change in lncRNA 74.1, NRG1, and miR-324-3p expression (Figure 6B). Western blot revealed that relative to oe-NC + DMSO, cells treated with oe-lncRNA 74.1 + DMSO showed enhanced NRG1 expression and reduced p-PI3K and p-AKT expression, while those treated with si-lncRNA 74.1 + DMSO showed opposite results. Compared with oe-lncRNA 74.1 + DMSO, the cells treated with oe-lncRNA 74.1 + SC79 exhibited elevated p-AKT expression. Compared with si-lncRNA 74.1 + DMSO, treatment with si-lncRNA 74.1 + SC79 elevated p-AKT expression. No significant difference in AKT and PI3K expression was found after comparison (Figure 6C).

Furthermore, it was observed that, compared with HK-2 cells treated with oe-NC + DMSO, the protein levels of ATG5, ATG7, and LC3II/I were significantly increased, while the P62 protein level was markedly reduced in the cells treated with si-lncRNA 74.1 + DMSO. Compared with oe-lncRNA 74.1 + DMSO treatment, protein levels of ATG5, ATG7, and LC3II/I were decreased, while the P62 protein level was markedly increased in the cells treated with oe-lncRNA 74.1 + SC79. Compared with si-lncRNA 74.1 + DMSO treatment, cells treated with si-lncRNA 74.1 + SC79 had reduced protein levels of ATG5, ATG7, and LC3II/I, but enhanced P62 protein level (Figure 6D). Figure 6E illustrates the results of TEM observation, demonstrating that treatment with oe-lncRNA 74.1 + DMSO resulted in increased number of autophagosomes compared with oe-NC + DMSO, whereas si-lncRNA 74.1 + DMSO treatment reduced the number of autophagosomes. Compared with oe-lncRNA 74.1 + DMSO, oe-lncRNA74.1 + SC-79 treatment led to reduced number of autophagosomes. Compared with si-lncRNA 74.1 + DMSO treatment, cells treated with si-lncRNA 74.1 + SC79 had a reduced number of autophagosomes. In addition, immunofluorescence demonstrated an elevated proportion of LC3 positive cells in the HK-2 cells following oe-lncRNA 74.1 + DMSO treatment, versus a reduced proportion of LC3 positive cells after si-lncRNA 74.1 + DMSO treatment compared to those treated with oe-NC + DMSO. A decreased proportion of LC3 positive cells was found in cells treated with oe-lncRNA 74.1 + SC79 compared with oe-lncRNA 74.1 + DMSO treatment. Compared with si-lncRNA 74.1 + DMSO treatment, cells treated with si-lncRNA 74.1 + SC79 presented with a reduced proportion of LC3 positive cells (Figure 6F). Additionally, as shown in Figure 6G, HK-2 cells treated with oe-lncRNA 74.1 + DMSO exhibited decreased levels of Collagen I, Fibronectin, and α-SMA, while si-lncRNA 74.1 + DMSO treatment exhibited elevated levels of Collagen I, Fibronectin, and α-SMA compared to cells treated with oe-NC + DMSO. Next, increased levels were observed in cells treated with oe-lncRNA 74.1 + SC79 compared with oe-lncRNA 74.1 + DMSO treatment. Compared with si-lncRNA 74.1 + DMSO treatment, cells treated with si-lncRNA 74.1 + SC79 presented with enhanced levels of Collagen I, Fibronectin, and α-SMA. These results suggested that lncRNA 74.1 could promote NRG1 expression through miR-324-3p to inhibit activation of the PI3K/AKT signaling pathway, thus inducing cell autophagy and inhibiting renal fibrosis.

### LncRNA 74.1 Inhibits Renal Fibrosis *via* the miR-324-3p/NRG1 Axis in a Unilateral Ureteral Obstruction Mouse Model

**FIGURE 7:**
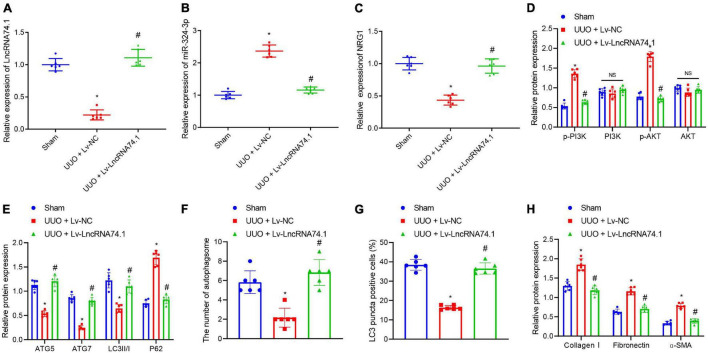
The *in vivo* verification of lncRNA 74.1 inhibition of renal fibrosis by inhibition of miR-324-3p/NRG1-mediated PI3K/AKT signaling pathway. **(A)** lncRNA 74.1 expression in mouse renal tissues determined using RT-qPCR; **(B)** miR-324-3p expression in mouse renal tissues determined using RT-qPCR; **(C)** NRG1 expression in mouse renal tissues determined using RT-qPCR; **(D)** protein expressions of PI3K, p-PI3K, AKT, and p-AKT in mouse renal tissues by Western blot; **(E)** protein expressions of ATG5, ATG7, and LC3II/I in mouse renal tissues by Western blot; **(F)** TEM observation of autophagosomes in mouse renal tissues with different treatments; **(G)** proportion of LC3 positive cells in mouse renal tissues with different treatments by immunofluorescence assay; **(H)** protein level of fibrosis markers in in mouse renal tissues by Western blot. *p < 0.05 as compared with the sham group; ^#^*p* < 0.05 as compared with the UUO + Lv-NC group. The measurement data were expressed as mean ± standard deviation. One-way ANOVA was used for data comparisons among multiple groups, followed by Tukey’s *post hoc* test. *n* = 6.

The expression of lncRNA 74.1, miR-324-3p, and NRG1 were detected in the kidney tissues of mice in each group by means of RT-qPCR, which revealed that, compared with the sham group, lncRNA 74.1 and NRG1 expression was lower, while that of miR-324-3p was higher in the UUO + Lv-NC group. In comparison with the UUO + Lv-NC group, lncRNA 74.1 and NRG1 expression was found to be higher, while that of miR-324-3p was lower in the UUO + Lv-lncRNA 74.1 group (Figures 7A–C). Western blot showed that UUO + Lv-NC treatment induced elevated p-AKT and p-AKT levels comparing to sham operation. UUO + Lv-lncRNA 74.1 treatment showed reduced p-AKT and p-AKT levels comparing to UUO + Lv-NC treatment (Figure 7D and Supplementary Figure 8C). In addition, UUO + Lv-NC treatment showed reduced ATG5, ATG7, and LC3II/I levels, and increased P62 level comparing to sham operation, while UUO + Lv-lncRNA 74.1 treatment led to increased ATG5, ATG7, and LC3II/I levels, and reduced P62 level comparing to UUO + Lv-NC treatment (Figure 7E).

Meanwhile, TEM observation showed that, compared with the sham group, the UUO + Lv-NC group presented with a reduced number of autophagosomes, while compared with the UUO + Lv-NC group, an increased number of autophagosomes were noted in the UUO + Lv-lncRNA 74.1 group (Figure 7F and Supplementary Figure 6C). Furthermore, immunofluorescence results depicted a reduced proportion of LC3 positive cells in the UUO + Lv-NC group compared with the sham group, along with an increased proportion of LC3 positive cells in the UUO + Lv-lncRNA 74.1 group compared with the UUO + Lv-NC group (Figure 7G and Supplementary Figure 7C). Finally, Western blot displayed that the levels of Collagen I, Fibronectin, and α-SMA were all increased in the UUO + Lv-NC group compared with the sham group, while decreased in the UUO + Lv-lncRNA 74.1 group compared with the UUO + Lv-NC group (Figure 7H).

Elevated plasma levels of creatinine are an important clinical indicator of impaired renal function. Therefore, we further detected the content of plasma creatinine in in the mouse model. The results showed that the content of creatinine in the UUO + Lv-NC group was increased compared with the sham group. Compared with the UUO + Lv-NC group, the plasma creatinine level in mice in the UUO + Lv-lncRNA 74.1 group was significantly lower (Supplementary Figure 9).

The above results showed that lncRNA 74.1 upregulated NRG1 through targeted adsorption of miR-324-3p, thus inhibiting PI3K/AKT signaling pathway, and then inducing autophagy to alleviate renal fibrosis in UUO mice.

## Discussion

**FIGURE 8:**
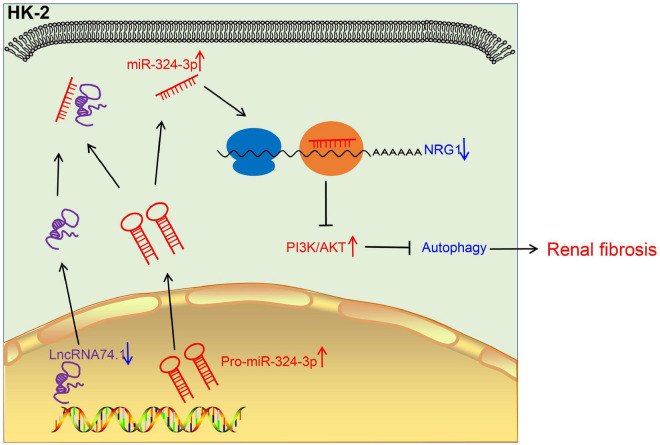
The mechanistic diagram illustrating the role of lncRNA 74.1/miR-324-3p/NRG1-mediated PI3K/AKT signaling pathway in renal fibrosis. In renal fibrosis, lncRNA 74.1 expressed poorly and miR-324-3p expressed highly. LncRNA 74.1 could bind to miR-324-3p and negatively regulate its expression, which further promoted the NRG1 expression and inactivated the PI3K/AKT signaling pathway, thereby inducing cellular autophagy, and inhibiting renal fibrosis.

Renal fibrosis can eventually progress to loss of normal physiological functions of the kidney, severely impacting the quality of life of patients. However, the limited currently available treatments fail to relieve completely this disease (Sun et al., 2017). Hence, there is an urgent need to identify the potential mechanisms of the regulation of renal fibrosis. Autophagy is a complex and systematic hemostatic process serving to eliminate the dysfunctional intracellular contents *via* a lysosome-dependent pathway (Sun et al., 2017). Existing knowledge further indicates that the process of autophagy holds the potential to promote cell survival as well as to augment the rapid removal of impaired organelles in cells, such as in the case of collagen secreted by myofibroblasts. Moreover, autophagy can improve the intracellular degradation of type I collagen mediated by TGF-β1 (He et al., 2014). Our findings demonstrated that lncRNA 74.1 could induce the autophagy of renal tubular epithelial cells to comprehensively inhibit renal fibrogenesis. In addition, lncRNA 74.1 specifically bound to miR-324-3p and then stimulated NRG1 expression, which suppressed the renal fibrosis mediated by the PI3K/AKT signaling pathway (Figure 8).

Initial findings obtained in the current study demonstrated that lncRNA 74.1 could bind to miR-324-3p to inhibit the fibrogenesis of kidney tissues. Indeed, various studies have highlighted the ability of lncRNA 74.1 to facilitates the reduction of extracellular matrix *via* autophagy to alleviate renal fibrosis (Xiao et al., 2019). However, the exact molecular mechanism underlying the regulation of renal fibrosis by lncRNA 74.1 was unknown. Combined with our previous research (Xiao et al., 2019), we speculated that lncRNA 74.1 could induce cellular autophagy in kidney, and that the upregulated rate of apoptosis could alleviate renal fibrosis. On the other hand, the RNA–RNA interaction between lncRNA and miRNAs is known to play a critical role in regulating the transcription of corresponding coding genes in disease development. Similar regulation patterns were observed in the study performed by Zhang et al., wherein lncRNA NR_038323 was found to sponge miR-324-3p to suppress radically renal fibrosis in the case of diabetic nephropathy (Ge et al., 2019). Meanwhile, miR-324-3p has been documented to function as an oncogene in various types of cancers, such as hepatocellular carcinoma, gastric cancer, and colon cancer (Tuo et al., 2017; Sun et al., 2018). Our findings were consistent with those observations, and indicated the functionality of miR-324-3p as a boosting factor to induce renal fibrosis. Furthermore, another study documented the clinical significance of miR-324-3p as a tumor suppressor in nasopharyngeal carcinoma, which indicates that miR-324-3p can serve varying roles depending upon the pathology or malignancy (Zhang et al., 2017). Expanding on our existing knowledge, our present findings further revealed that NRG-1 was specifically-targeted by miR-324-3p in renal fibrosis, which was further coordinated by lncRNA 74.1, indicating that overexpression of NRG-1 could significantly reduce the fibrogenesis of renal epithelial cells. An endothelium-derived growth factor, NRG-1 is widely-known to be implicated in a plethora of diseases, with studies even highlighting the participation of NRG-1 in the kidney disorders (Zhang et al., 2016; Yang et al., 2017; Gunadi et al., 2018). Furthermore, systemic administration of rhNRG-1 in hypercholesterolemic type 1 diabetic mice protected simultaneously against complications in the heart, arteries, and kidneys (Vandekerckhove et al., 2016). Nevertheless, the underlying mechanism of the aforementioned protective role of NRG-1 requires extensive investigation. More notably, inhibition of miR-223-5p brought about a suppressed inflammatory response and augmented NRG-1 levels to reduce glia reaction and neuron apoptosis in brain, which is very similar to the direct-targeting relationship btw miR-324-3p and NRG-1 observed in our study (Guan et al., 2019). Moreover, studies have also documented that miR-143 directly-targets to the 3′ un-translated region of NRG-1 mRNA to reduce the expression of NRG1 (Wang P. et al., 2019). Consequently, it would be plausible to suggest that NRG-1 is specifically-targeted by miR-324-3p. Altogether, the aforementioned data indicate that lncRNA 74.1 regulates miR-324-3p, which further targets and inhibits the expression of NRG1, and that lncRNA 74.1 antagonized the inhibitory effect of miR-324-3p on the expression of NRG1.

Furthermore, our findings also revealed that the PI3K/AKT signaling pathway mediated the regulation of renal fibrosis *via* lncRNA 74.1. The PI3K/AKT signaling pathway is critical downstream response factor to TGF-β, which is widely-regarded as a central regulator of renal fibrosis (Meng et al., 2016). Akt possesses the ability to drive the matrix protein expression mediated by TGF-β signal induced mTOR (Das et al., 2018). Moreover, accumulating evidence has shown that PI3K/AKT signaling pathway can also regulate renal fibrosis in multiple animal and cellular models. For instance, Nrf2 signaling was previously noted to regulate the PI3K/AKT signaling pathway, which subsequently attenuated epithelial-to-mesenchymal transition (EMT) and renal interstitial fibrosis (Wang J. et al., 2019). Similarly, hypoxia-induced Bmi1 can promote the manifestation of EMT and renal fibrosis through PI3K/AKT signaling pathway (Du et al., 2014). Meanwhile, the anthraquinone compound Aloe-Emodin has been previously illustrated to suppress the fibrogenesis of kidney cells *in vitro* and *in vivo* by inhibiting the PI3K/AKT/mTOR signaling pathway (Dou et al., 2019). From the aforementioned data, it can be concluded that the PI3K/AKT signaling pathway regulation is a vital mediator in the progression of renal fibrosis.

## Conclusion

Collectively, our findings elicited a new axis of lncRNA 74.1/miR-324-3p/NRG1/PI3K/AKT in the regulation of fibrogenesis development in renal tubular epithelial cells. However, our study still presents with certain limitations regarding the relationship between EMT and renal fibrosis mediated by TGF-β signaling and how NRG1 might affects PI3K/AKT signaling pathway. Nevertheless, our efforts have successfully illustrated lncRNA 74.1 sponging of miR-324-3p as a key regulator of fibrosis, which provides a new insight for the future development of RNA medication to treat kidney fibrosis.

## Data Availability Statement

The original contributions presented in the study are included in the article/Supplementary Material, further inquiries can be directed to the corresponding author.

## Ethics Statement

The studies involving human participants were reviewed and approved by Xiangya Hospital, Central South University. The patients/participants provided their written informed consent to participate in this study. The animal study was reviewed and approved by Xiangya Hospital, Central South University.

## Author Contributions

ST: conceptualization and writing—review and editing. GX: methodology and validation. QY: data curation and formal analysis. WL: formal analysis and roles/writing—original draft. XY: investigation and software. XF: conceptualization and roles/writing—original draft. TD: resources and writing—review and editing. XX: conceptualization and project administration. All authors contributed to the article and approved the submitted version.

## Conflict of Interest

The authors declare that the research was conducted in the absence of any commercial or financial relationships that could be construed as a potential conflict of interest.

## Publisher’s Note

All claims expressed in this article are solely those of the authors and do not necessarily represent those of their affiliated organizations, or those of the publisher, the editors and the reviewers. Any product that may be evaluated in this article, or claim that may be made by its manufacturer, is not guaranteed or endorsed by the publisher.

## Supplementary Material

Supplementary Figure 1 Representative HE staining results of 6 normal specimens, 11 mild fibrosis specimens, and 13 moderate fibrosis specimens. Scale bar = 50 μm.

Supplementary Figure 2 The changes of LC3II/I ratio in cells of each group were detected by Western blot. ^∗^*p* < 0.05. The measurement data were expressed as mean ± standard deviation. Data between the two groups were compared using the unpaired *t-*test. Each experiment was repeated six times independently.

Supplementary Figure 3 Intersection miRNA regulated by lncRNA 74.1 predicted using miRanda.

Supplementary Figure 4 RT-qPCR of transfection efficacies of miR-324-3p and lncRNA 74.1 as well as effect of lncRNA 74.1 interference on the expression of miR-324-3p. **(A)** efficacy of overexpressed or silenced miR-324-3p in HEK293T cells and HK-2 cells by RT-qPCR; **(B)** RT-qPCR results of miR-324-3p and lncRNA 74.1 expression after lncRNA 74.1 overexpression or silencing. ^∗^*p* < 0.05 as compared with mimic-NC or oe-NC; ^#^*p* < 0.05 as compared with inhibitor-NC or si-NC. The measurement data were expressed as mean ± standard deviation. Data between the two groups were compared using the unpaired *t-*test. Each experiment was repeated three times independently.

Supplementary Figure 5 The expression of miR-324-3p after lncRNA74.1 was silenced in HK-2 cells. ^∗^*p* < 0.05 as compared with si-NC. The measurement data were expressed as mean ± standard deviation. Data between the two groups were compared using the unpaired *t-*test. Each experiment was repeated three times independently.

Supplementary Figure 6 TEM observation. **(A)** TEM observation of autophagosomes in HK-2 cells with different treatments; **(B)** TEM observation of autophagosomes in HK-2 cells with different treatments; **(C)** TEM observation of autophagosomes in mouse renal tissues with different treatments.

Supplementary Figure 7 Immunofluorescence pictures. **(A)** proportion of LC3 positive cells in the HK-2 cells with different treatments by immunofluorescence assay; **(B)** proportion of LC3 positive cells in the HK-2 cells with different treatments by immunofluorescence assay; **(C)** proportion of LC3 positive cells in mouse renal tissues with different treatments by immunofluorescence assay.

Supplementary Figure 8 Western blots. **(A)** protein level of fibrosis markers in HK-2 cells with different treatments by Western blot; **(B)** Western blot results of NRG1 protein expression in HK-2 cells with different treatments; **(C)** protein expressions of PI3K, p-PI3K, AKT, and p-AKT in mouse renal tissues by Western blot.

Supplementary Figure 9 Determination of creatinine in mouse plasma under different treatment conditions. ^∗^*p* < 0.05 as compared with sham, ^#^*p* < 0.05 as compared with UUO + Lv-NC. The measurement data were expressed as mean ± standard deviation. Data between the two groups were compared using the unpaired *t-*test. Each experiment was repeated three times independently.
