# Supplementary figures and images for: Long Non-coding RNA ENST00000453774.1 Confers an Inhibitory Effect on Renal Fibrosis by Inhibiting miR-324-3p to Promote NRG1 Expression
Source: Front Cell Dev Biol. 2021 Nov 19;9:580754. doi: 10.3389/fcell.2021.580754 (PMC8640469; doi:10.3389/fcell.2021.580754)

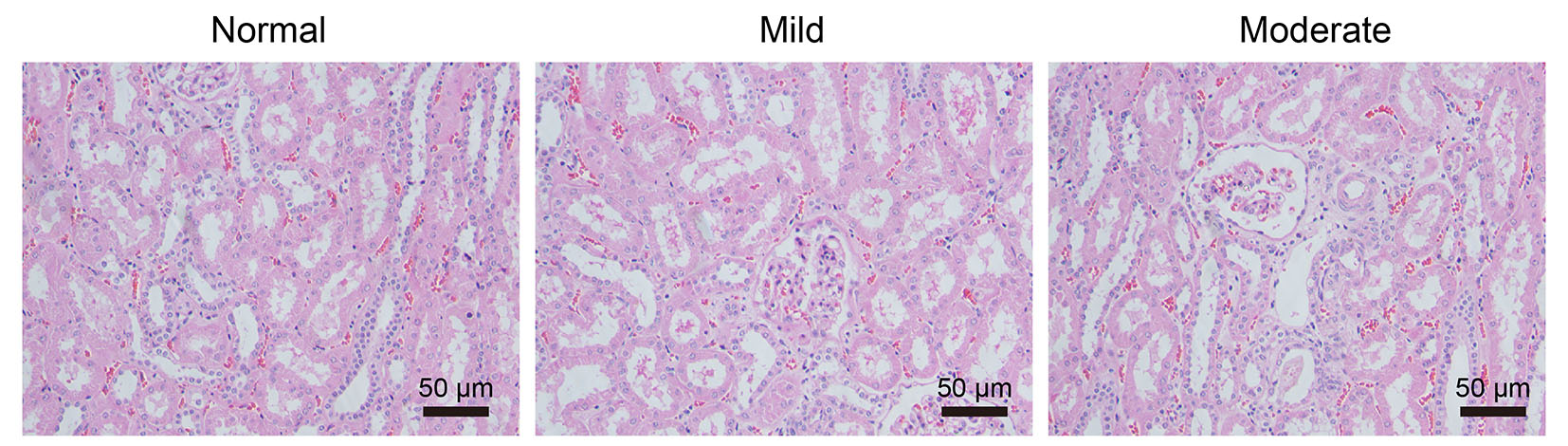

Supplement: Supplementary Figure 1 — Representative HE staining results of 6 normal specimens, 11 mild fibrosis specimens, and 13 moderate fibrosis specimens. Scale bar = 50 μm. [file Image_1.JPEG]

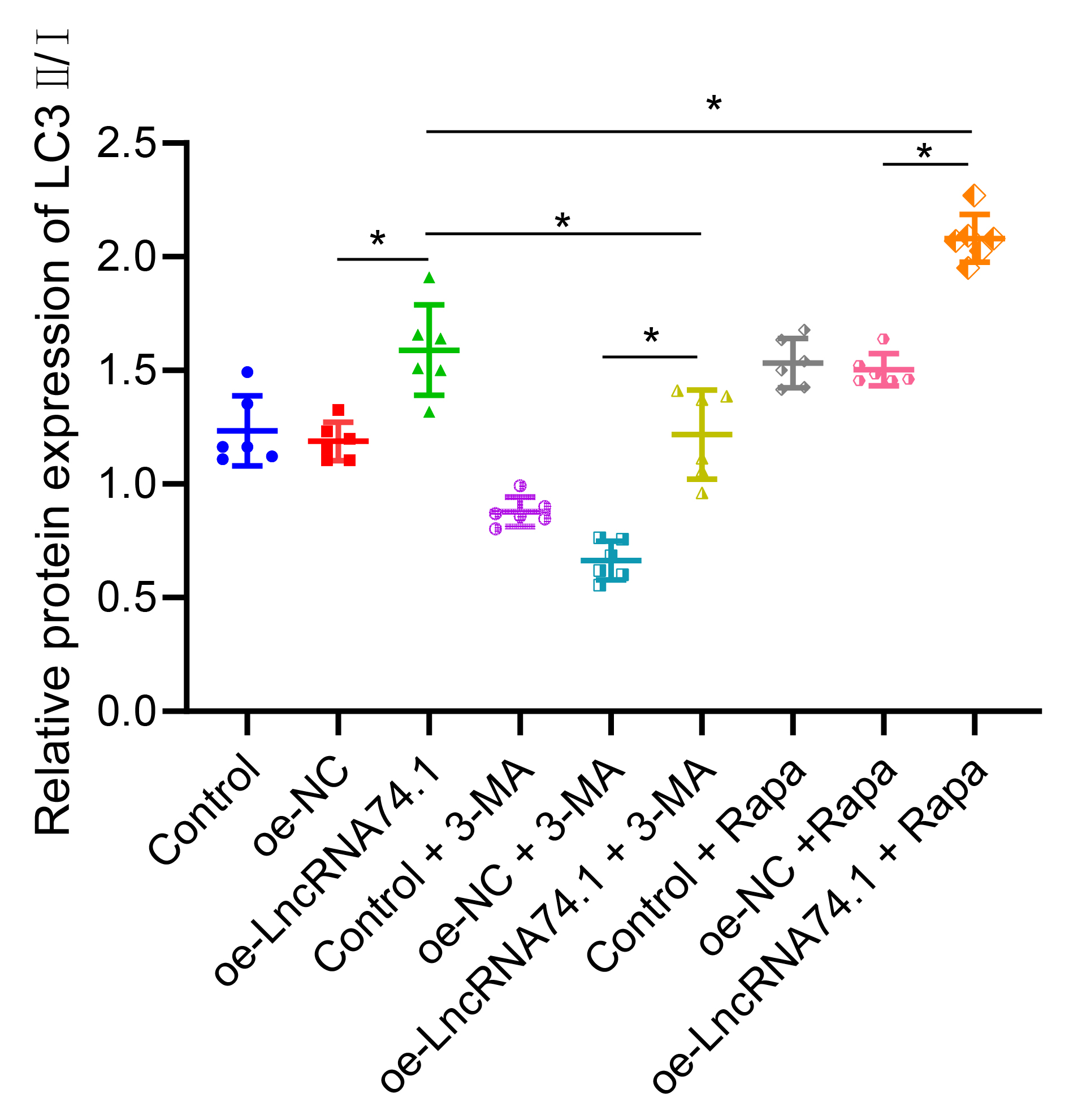

Supplement: Supplementary Figure 2 — The changes of LC3II/I ratio in cells of each group were detected by Western blot. ∗p < 0.05. The measurement data were expressed as mean ± standard deviation. Data between the two groups were compared using the unpaired t-test. Each experiment was repeated six times independently. [file Image_2.jpg]

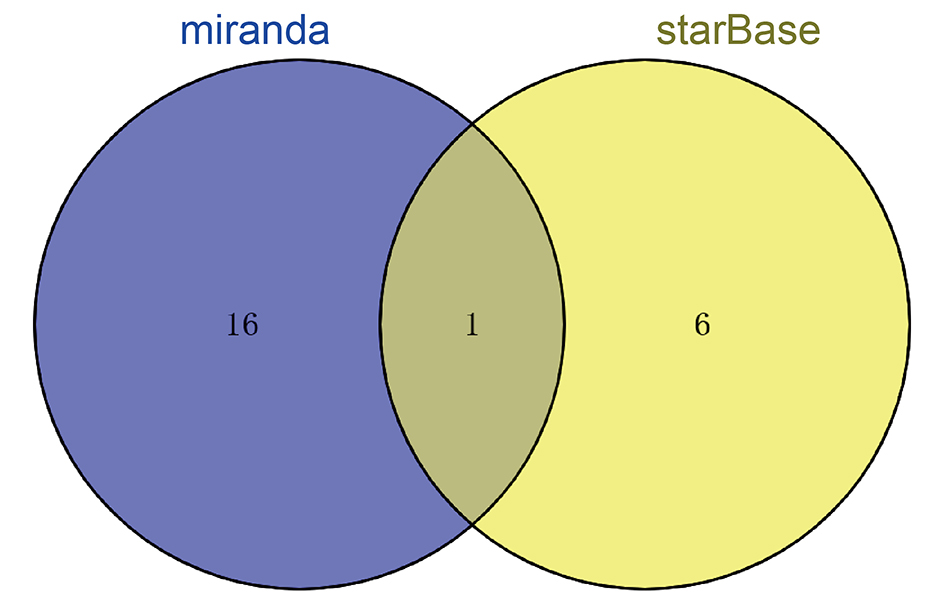

Supplement: Supplementary Figure 3 — Intersection miRNA regulated by lncRNA 74.1 predicted using miRanda. [file Image_3.JPEG]

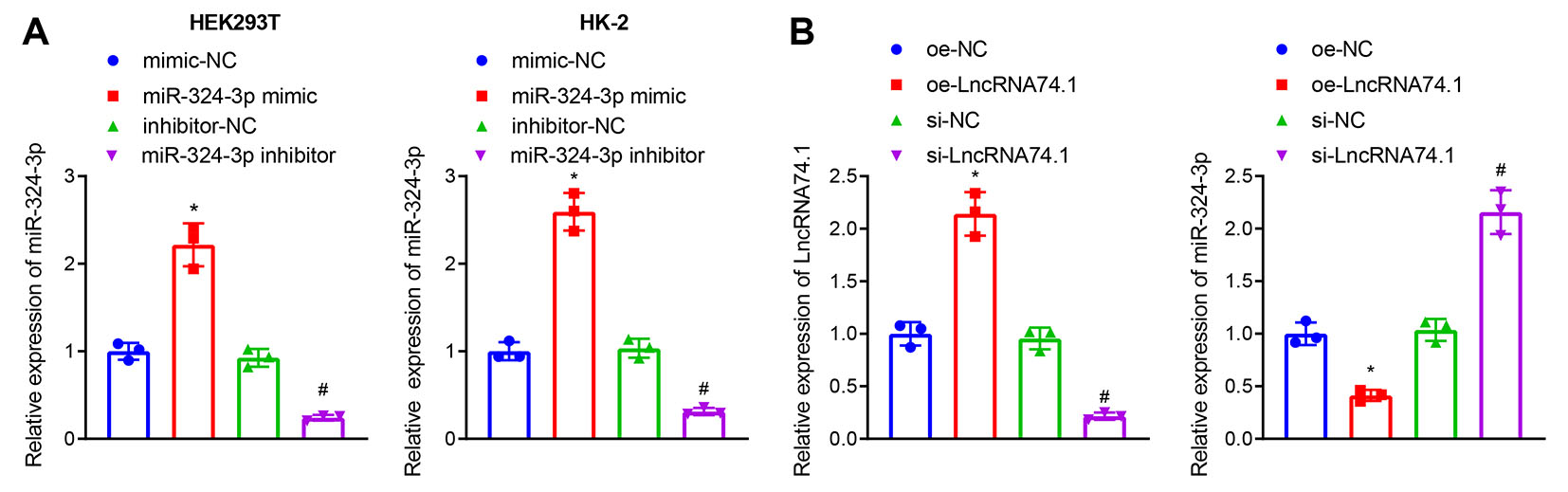

Supplement: Supplementary Figure 4 — RT-qPCR of transfection efficacies of miR-324-3p and lncRNA 74.1 as well as effect of lncRNA 74.1 interference on the expression of miR-324-3p. (A) efficacy of overexpressed or silenced miR-324-3p in HEK293T cells and HK-2 cells by RT-qPCR; (B) RT-qPCR results of miR-324-3p and lncRNA 74.1 expression after lncRNA 74.1 overexpression or silencing. ∗p < 0.05 as compared with mimic-NC or oe-NC; #p < 0.05 as compared with inhibitor-NC or si-NC. The measurement data were expressed as mean ± standard deviation. Data between the two groups were compared using the unpaired t-test. Each experiment was repeated three times independently. [file Image_4.JPEG]

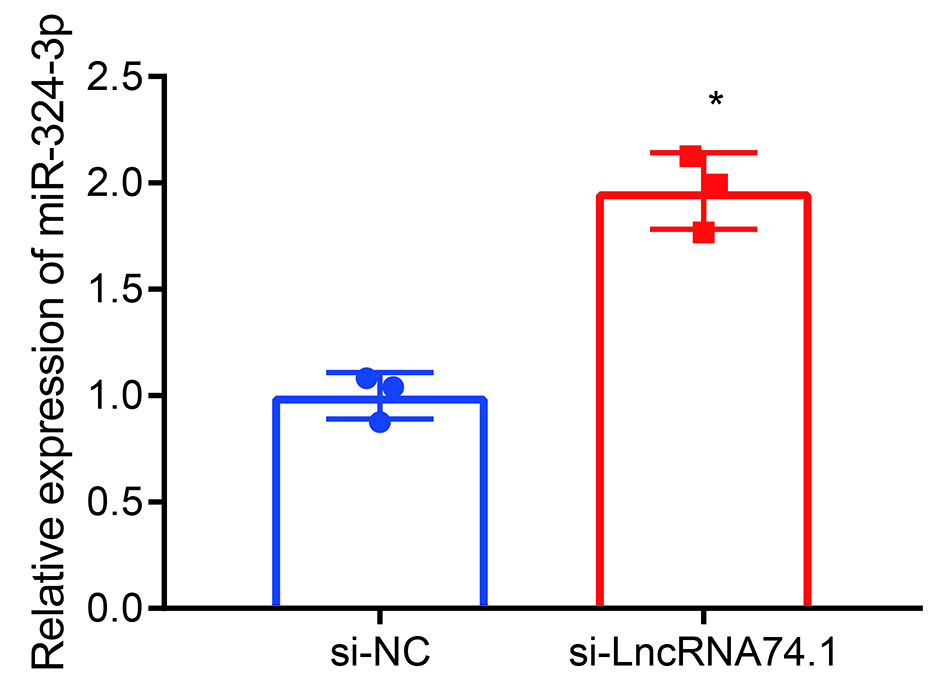

Supplement: Supplementary Figure 5 — The expression of miR-324-3p after lncRNA74.1 was silenced in HK-2 cells. ∗p < 0.05 as compared with si-NC. The measurement data were expressed as mean ± standard deviation. Data between the two groups were compared using the unpaired t-test. Each experiment was repeated three times independently. [file Image_5.JPEG]

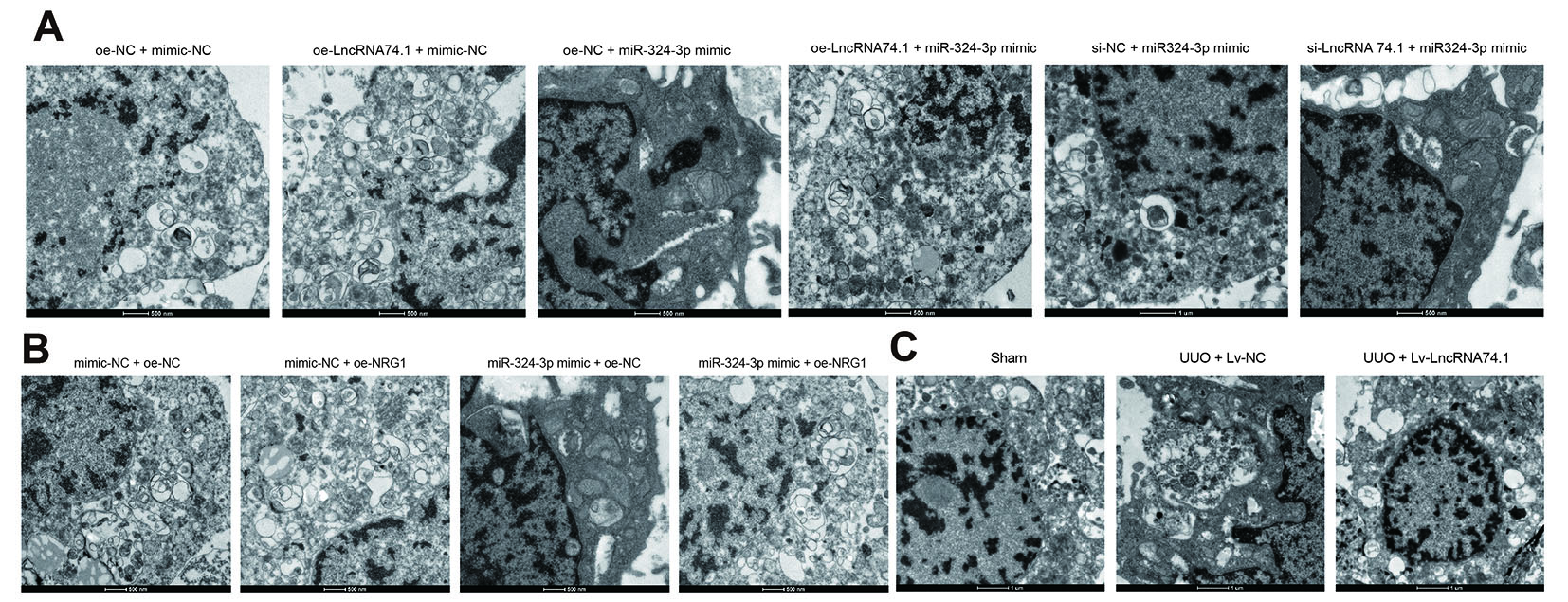

Supplement: Supplementary Figure 6 — TEM observation. (A) TEM observation of autophagosomes in HK-2 cells with different treatments; (B) TEM observation of autophagosomes in HK-2 cells with different treatments; (C) TEM observation of autophagosomes in mouse renal tissues with different treatments. [file Image_6.JPEG]

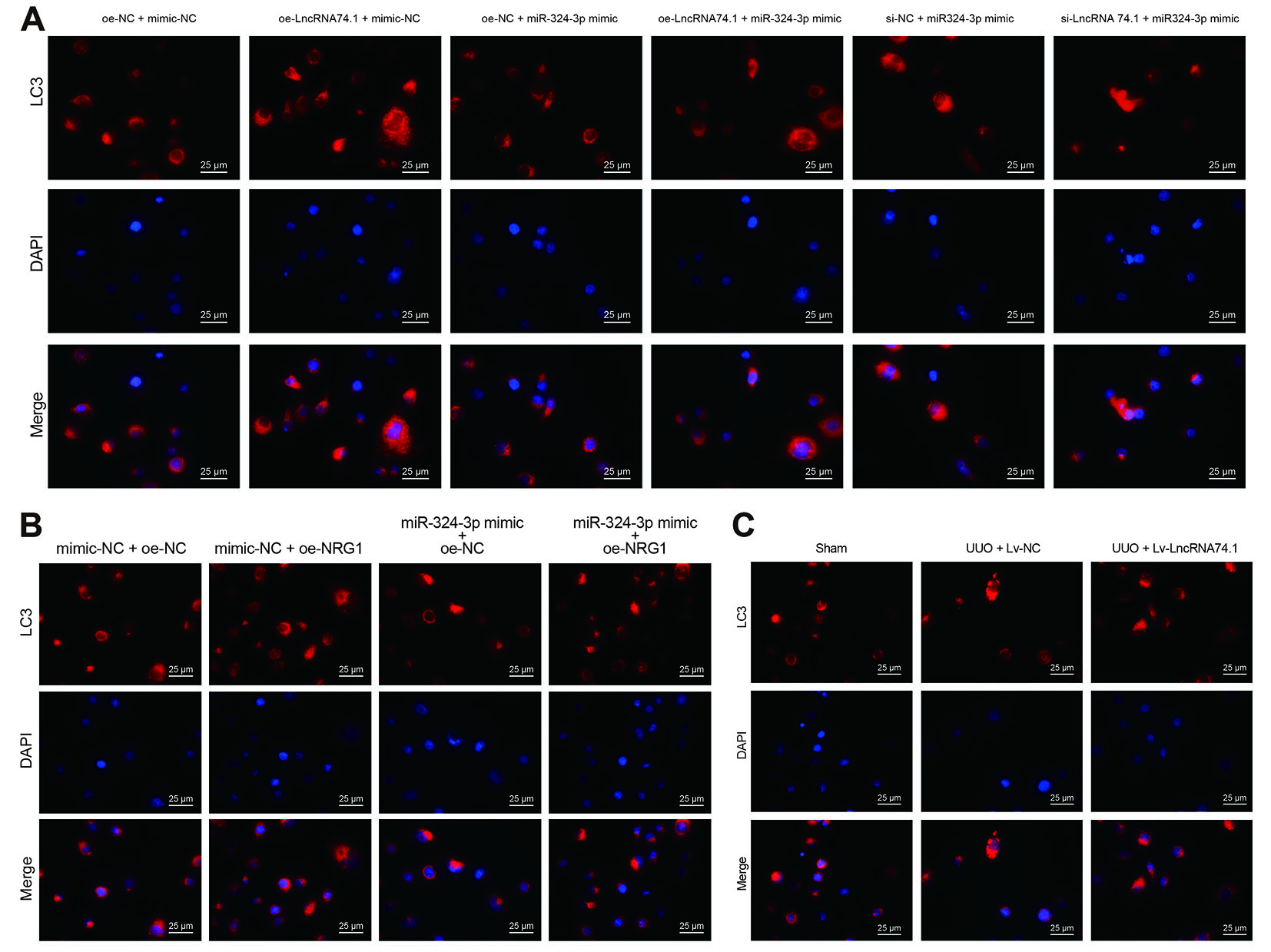

Supplement: Supplementary Figure 7 — Immunofluorescence pictures. (A) proportion of LC3 positive cells in the HK-2 cells with different treatments by immunofluorescence assay; (B) proportion of LC3 positive cells in the HK-2 cells with different treatments by immunofluorescence assay; (C) proportion of LC3 positive cells in mouse renal tissues with different treatments by immunofluorescence assay. [file Image_7.JPEG]

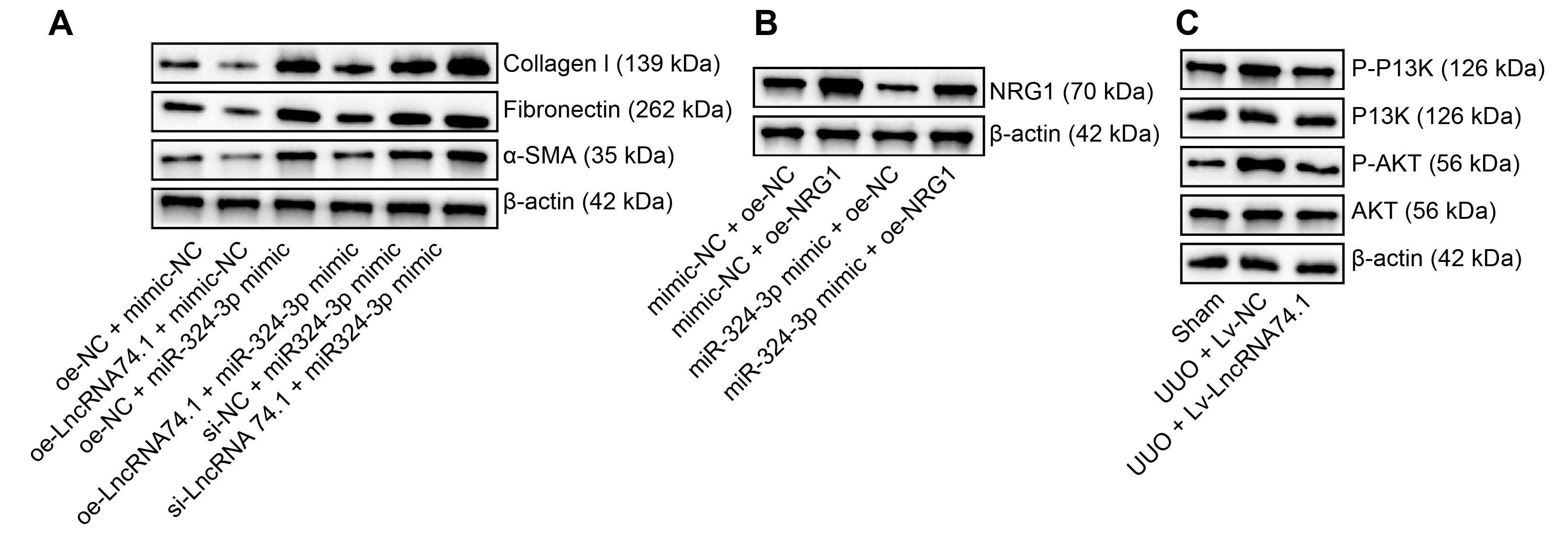

Supplement: Supplementary Figure 8 — Western blots. (A) protein level of fibrosis markers in HK-2 cells with different treatments by Western blot; (B) Western blot results of NRG1 protein expression in HK-2 cells with different treatments; (C) protein expressions of PI3K, p-PI3K, AKT, and p-AKT in mouse renal tissues by Western blot. [file Image_8.jpg]

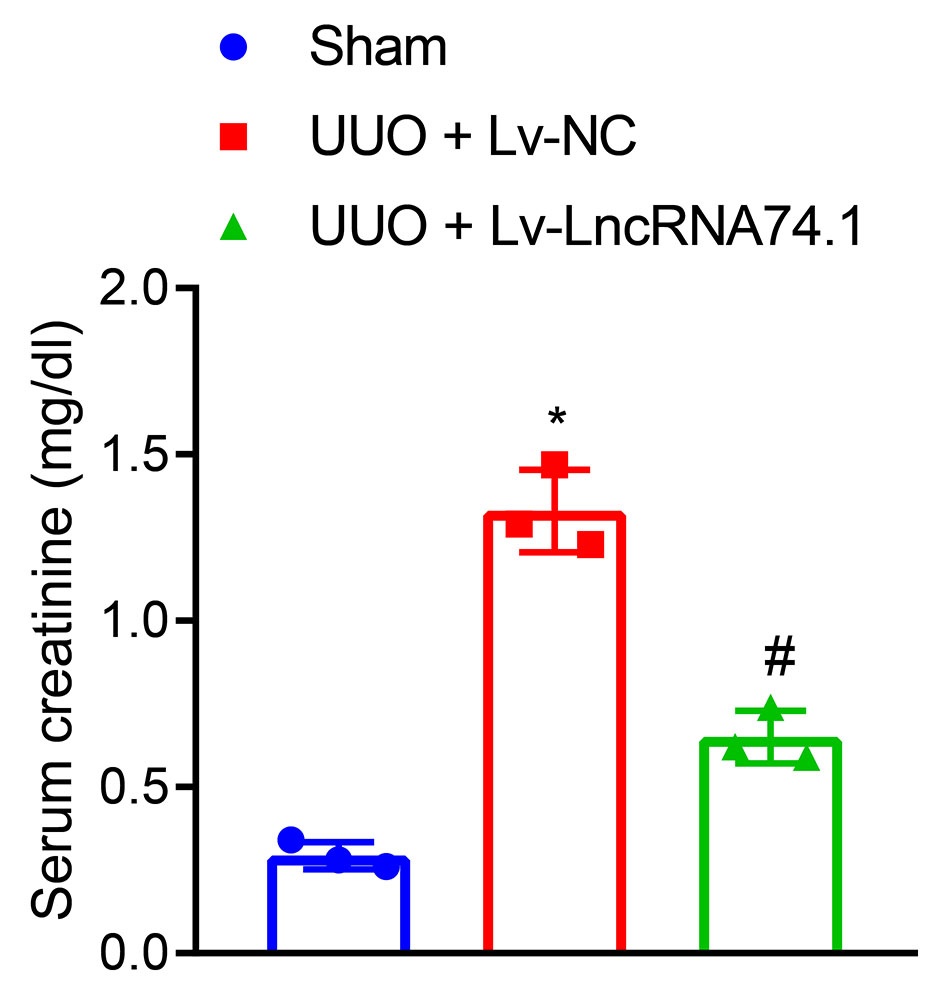

Supplement: Supplementary Figure 9 — Determination of creatinine in mouse plasma under different treatment conditions. ∗p < 0.05 as compared with sham, #p < 0.05 as compared with UUO + Lv-NC. The measurement data were expressed as mean ± standard deviation. Data between the two groups were compared using the unpaired t-test. Each experiment was repeated three times independently. [file Image_9.JPEG]
