# Supplementary material for: Long Non-coding RNA ENST00000453774.1 Confers an Inhibitory Effect on Renal Fibrosis by Inhibiting miR-324-3p to Promote NRG1 Expression
Source: Front Cell Dev Biol. 2021 Nov 19;9:580754. doi: 10.3389/fcell.2021.580754 (PMC8640469; doi:10.3389/fcell.2021.580754)
Supplement: Supplementary file 10 [file Table_1.DOCX]

**Table S1.** Primer sequences for reverse transcription quantitative polymerase chain reaction

| Genes | Primer sequences (5’ - 3’) |
| --- | --- |
| LncRNA 74.1 | Forward: ATGTGGAGAAGCGTCAGA  Reverse: CCATTACATGATTTTATAGG |
| miR-324-3p | Forward: CGGCGGACTGCCCCAGGTGC  Reverse: Universal reverse primers |
| NRG1 | Forward: CGGTGTCCATGCCTTCCAT  Reverse: GTGTCACGAGAAGTAGAGGTCT |
| GAPDH | Forward: ACCACAGTCCATGCCATCAC  Reverse: CCACCACCCTGTTGCTGTAG |
