# Supplementary material for: Long Non-coding RNA ENST00000453774.1 Confers an Inhibitory Effect on Renal Fibrosis by Inhibiting miR-324-3p to Promote NRG1 Expression
Source: Front Cell Dev Biol. 2021 Nov 19;9:580754. doi: 10.3389/fcell.2021.580754 (PMC8640469; doi:10.3389/fcell.2021.580754)
Supplement: Supplementary file 11 [file Table_2.DOCX]

**Table S2.** miRNAs could bind to lncRNA74.1

| Seq1 | Seq2 | Tot Score | Tot Energy | Max Score | Max Energy |
| --- | --- | --- | --- | --- | --- |
| hsa-miR-324-3p | ENST00000453774 | 164 | -31.3 | 164 | -31.3 |
| hsa-miR-432-5p | ENST00000453774 | 167 | -25.13 | 167 | -25.13 |
| hsa-miR-514a-5p | ENST00000453774 | 167 | -24.82 | 167 | -24.82 |
| hsa-miR-548k | ENST00000453774 | 162 | -11.52 | 162 | -11.52 |
| hsa-miR-1269a | ENST00000453774 | 303 | -44.15 | 157 | -24.27 |
| hsa-miR-3121-3p | ENST00000453774 | 309 | -27.62 | 157 | -14.33 |
| hsa-miR-4294 | ENST00000453774 | 287 | -34.98 | 145 | -20.13 |
| hsa-miR-4282 | ENST00000453774 | 161 | -11.32 | 161 | -11.32 |
| hsa-miR-3135b | ENST00000453774 | 302 | -40.44 | 153 | -21.63 |
| hsa-miR-1269b | ENST00000453774 | 303 | -43.06 | 157 | -24.27 |
| hsa-miR-4529-3p | ENST00000453774 | 300 | -34.48 | 160 | -20.49 |
| hsa-miR-4531 | ENST00000453774 | 293 | -24.24 | 149 | -12.17 |
| hsa-miR-4713-3p | ENST00000453774 | 302 | -42.1 | 157 | -21.1 |
| hsa-miR-4715-3p | ENST00000453774 | 294 | -35.6 | 152 | -19.66 |
| hsa-miR-4727-3p | ENST00000453774 | 166 | -23.44 | 166 | -23.44 |
| hsa-miR-5186 | ENST00000453774 | 299 | -32.56 | 150 | -17.1 |
| hsa-miR-6730-5p | ENST00000453774 | 168 | -25.33 | 168 | -25.33 |
