# Supplementary material for: Long Non-coding RNA ENST00000453774.1 Confers an Inhibitory Effect on Renal Fibrosis by Inhibiting miR-324-3p to Promote NRG1 Expression
Source: Front Cell Dev Biol. 2021 Nov 19;9:580754. doi: 10.3389/fcell.2021.580754 (PMC8640469; doi:10.3389/fcell.2021.580754)
Supplement: Supplementary file 12 [file Table_3.DOCX]

**Table S3.** miRNAs could bind to lncRNA74.1 predicted by Starbase database

| miRNAid | miRNAname | geneName | geneType | miRseq |
| --- | --- | --- | --- | --- |
| MIMAT0000435 | hsa-miR-143-3p | LINC01320 | lincRNA | CUCGAUGUCACGAAGUAGAGU |
| MIMAT0023713 | hsa-miR-6088 | LINC01320 | lincRNA | GCGGGGGGGCGAAGUAGAGA |
| MIMAT0019924 | hsa-miR-4770 | LINC01320 | lincRNA | UCGAUGUCACAGUAGAGU |
| MIMAT0007888 | hsa-miR-1913 | LINC01320 | lincRNA | ACCGUCGUCGCCUCCCCCGUCU |
| MIMAT0000762 | hsa-miR-324-3p | LINC01320 | lincRNA | GGUCGUCGUGGACCCCGUCA |
| MIMAT0002818 | hsa-miR-496 | LINC01320 | lincRNA | CUCUAACCGGUAC-AUUAUGAGU |
| MIMAT0002835 | hsa-miR-526b-5p | LINC01320 | lincRNA | UGUCUUUCACGAAGGGAGUUCUC |
